# Smp38 MAP Kinase Regulation in *Schistosoma mansoni*: Roles in Survival, Oviposition, and Protection Against Oxidative Stress

**DOI:** 10.3389/fimmu.2019.00021

**Published:** 2019-01-24

**Authors:** Lívia das Graças Amaral Avelar, Sandra Grossi Gava, Renata Heisler Neves, Mercedes Carolina Soares Silva, Neusa Araújo, Naiara Clemente Tavares, Assmaa El Khal, Ana Carolina Alves Mattos, José Roberto Machado-Silva, Guilherme Oliveira, Marina de Moraes Mourão

**Affiliations:** ^1^Instituto de Ciências Biológicas, Universidade Federal de Minas Gerais–UFMG, Belo Horizonte, Brazil; ^2^Instituto René Rachou, Fundação Oswaldo Cruz–FIOCRUZ, Belo Horizonte, Brazil; ^3^Faculdade de Ciências Médicas, Universidade do Estado do Rio de Janeiro, Rio de Janeiro, Brazil; ^4^Instituto Tecnológico Vale, Belém, Brazil

**Keywords:** *Schistosoma mansoni*, p38 MAPK, signaling pathways, development, oviposition, oxidative stress, gene expression

## Abstract

Eukaryotic protein kinases (ePKs) are good medical targets for drug development in different biological systems. ePKs participate in many cellular processes, including the p38 MAPK regulation of homeostasis upon oxidative stress. We propose to assess the role of Smp38 MAPK signaling pathway in *Schistosoma mansoni* development and protection against oxidative stress, parasite survival, and also to elucidate which target genes have their expression regulated by Smp38 MAPK. After a significant reduction of up to 84% in the transcription level by Smp38 MAPK gene knockdown, no visible phenotypic changes were reported in schistosomula in culture. The development of adult worms was tested *in vivo* in mice infected with the Smp38 knocked-down schistosomula. It was observed that Smp38 MAPK has an essential role in the transformation and survival of the parasites as a low number of adult worms was recovered. Smp38 knockdown also resulted in decreased egg production, damaged adult worm tegument, and underdeveloped ovaries in females. Furthermore, only ~13% of the eggs produced developed into mature eggs. Our results suggest that inhibition of the Smp38 MAPK activity interfere in parasites protection against reactive oxygen species. Smp38 knockdown in adult worms resulted in 80% reduction in transcription levels on the 10th day, with consequent reduction of 94.4% in oviposition *in vitro*. In order to search for Smp38 MAPK pathway regulated genes, we used an RNASeq approach and identified 1,154 DEGs in Smp38 knockdown schistosomula. A substantial proportion of DEGs encode proteins with unknown function. The results indicate that Smp38 regulates essential signaling pathways for the establishment of parasite homeostasis, including genes related to antioxidant defense, structural composition of ribosomes, spliceosomes, cytoskeleton, as well as, purine and pyrimidine metabolism pathways. Our data show that the Smp38 MAPK signaling pathway is a critical route for parasite development and may present attractive therapeutic targets for the treatment and control of schistosomiasis.

## Introduction

Protein kinases (PKs) have been developed as drug targets, with several inhibitors already registered for clinical trials (1). In different organisms, several cellular processes, including growth, metabolism, apoptosis, and immune responses are regulated by kinases of the family of mitogen-activated protein kinases (MAPKs) (2–4). MAPK signaling pathways are evolutionally highly conserved and respond to a variety of extracellular stimuli, such as growth factors and environmental stresses. The response induces the sequential phosphorylation and activation of other proteins, culminating in changes in the transcriptional profile (5).

MAPK signaling pathways are well-conserved and include members of four subfamilies: extracellular signal-regulated kinase (ERK), c-Jun N-terminal kinase (JNK), Nemo-like kinase (NLK), and p38 MAP kinase (6). In many organisms, the p38 subfamily has four isoforms identified (p38 α/β/γ/δ) that interact with a diverse number of regulatory mechanisms (7–9), many of which are related to stress responses, inflammation, and apoptosis (10, 11).

In *Schistosoma mansoni*, a parasite that causes schistosomiasis, were identified nine MAPKs, including representatives of the subfamilies ERK, p38, JNK, and, NLK, and only one member of the p38 MAPK subfamily (Smp38, Smp_133020) is present (12). It has been already shown that Smp38 acts on the ciliary movement regulation and in the early post-embryonic parasite development (13, 14). In addition, the attenuation of Smp38 activity reduces the release of gland components in response to light, temperature, and linoleic acid, being critical to the mechanisms of parasite penetration in the intermediate host (15).

To successfully establish in different environments and hosts, schistosomes have evolved a number of evasion mechanisms (16, 17). In this sense, PKs are a very important class of proteins since they are activated in response to several stimuli (18) and thus promote transcriptional changes responsible for the adaptation to the environments to which it is exposed.

In the host-parasite interaction, *Schistosoma mansoni* is exposed to diverse host humoral and cellular cytotoxic factors (19). Antioxidants enzymes produced by the parasite are an essential survival mechanism to neutralize the oxidative stress generated by its hosts (20). It has already been shown that antioxidant defenses are involved in cellular redox balance, thus contributing to parasite larval survival in their intermediate snail host, *Biomphalaria glabrata* (19).

In order to elucidate Smp38 roles in the host- *S. mansoni* parasite interaction and survival to the different milieu, here we contribute to the characterization of Smp38 pathway focusing in the schistosomula and adult stages. We describe the Smp38 requirement for parasite development in the murine model and *in vitro*, including its survival against oxidative stress. Further, we identified, at the transcriptional level, genes regulated by the Smp38 pathway.

## Materials and Methods

### Ethics Statement

Animal care and experimental protocols were reviewed and approved by the Ethics Committee for Animal Use (CEUA) of Oswaldo Cruz Foundation under licenses numbers LW13/13 and LW12/16. All experimental procedures were performed according to the Brazilian ethical guidelines (Law 11794/08).

### Parasite Materials

The *S. mansoni* LE strain is maintained throughout passages between hamsters and *Biomphalaria glabrata* hosts, in the “Lobato Paraense” snail facility at the René Rachou Institute—FIOCRUZ.

Schistosomula were obtained by mechanical transformation of cercariae as previously described (21) and cultured in Glasgow Minimum Essential Medium (Sigma-Aldrich, Germany) supplemented with 0,2 μM triiodothyronine (Sigma-Aldrich, Germany); 0.1% glucose; 0.1% lactalbumin (Sigma-Aldrich, Germany); 20 mM HEPES; 0.5% MEM vitamin solution (Gibco, USA); 5% Schneider's Insect Medium (Sigma-Aldrich, Germany); 0.5 μM Hypoxanthine (Sigma-Aldrich, Germany), 1 μM hydrocortisone (Sigma-Aldrich, Germany), 1% Penicillin/Streptomycin (Gibco, USA) and 2% heat-inactivated Fetal Bovine Serum (Gibco, USA).

Approximately 300 cercariae were subcutaneously inoculated in Golden hamsters (*Mesocricetus auratus*) for adult worm recovery. After 40 days, the hamsters were euthanized by overdose and perfused with a saline solution containing heparin (2,500 U/L) (22). After perfusion, males and females worms were manually separated when necessary. Adult worms were then cultured in RPMI 1640 medium (Gibco, USA) supplemented with 10% heat-inactivated Fetal Bovine Serum (Gibco, USA) and 2% Penicillin/Streptomycin (Gibco, USA).

### Amplification, Cloning, and Sequencing

The sequence of Smp38 MAPK (Smp_133020) was obtained from the *S. mansoni* database, GeneDB (http://www.genedb.org/Homepage/Smansoni). Primers to amplify the complete sequence, fragments for dsRNA synthesis and RT-qPCR were designed using the Primer 3 program (http://primer3.sourceforge.net). Primers designed for dsRNAs syntheses contain the T7 promoter sequence added to the 5′-end. Fragments of green-fluorescent protein (GFP, from pCRII plasmid vector) and *Discosoma* sp. mCherry fluorescent protein (GenBank AY678264) were used as non-schistosome RNAi controls.

A fragment corresponding to the complete coding sequence was amplified by PCR using primers described in the Table S1 and then cloned into the pCR2.1-TOPO vector. Sequencing was carried out with DYEnamic ET Dye Terminator Cycle Sequencing Kit for MegaBACE DNA Analysis Systems (Amersham Bioscience, UK) according to the manufacturer's instructions. The sequences generated were aligned using the multiple sequence alignment program ClustalW 2.0 (http://www.ebi.ac.uk/Tools/clustalw2/index.html).

### Double-Stranded RNAi Exposure

After Smp38 sequence verification, two Smp38 MAPK fragments encompassing two different regions of the CDS (Smp38.1, ranging from the nucleotide position 342 to 894 nt−553 bp and Smp38.2 from the position 463 to 698 nt – 236 bp) were amplified by PCR using specific primers containing the T7 promoter (Table S1). The unspecific controls, mCherry (711 bp), or GFP (360 bp) dsRNAs were also synthesized from fragments cloned in plasmids. Double-stranded RNAs (dsRNAs) were synthesized using the T7 RiboMAX Express RNAi System kit (Promega, USA) according to the supplier's protocol; the reactions were carried out overnight at 37°C. DsRNAs integrity was confirmed in 1% agarose gel electrophoresis.

Immediately after cercariae transformation, schistosomula were exposed to 100 nM of dsRNAs (Smp38.1, Smp38.2, or mCherry—unspecific control) in 24 well-plates containing 3,000 parasites. Cultures were incubated at 37°C, 5% CO_2_, and 95% humidity with 2 mL of supplemented MEM medium. After two, four and 7 days of dsRNA exposure, 1,000 schistosomula were removed for relative expression evaluation using quantitative real-time PCR (RT-qPCR).

Electroporation of 25 μg of dsRNAs was used for adult worms RNAi assessment. Adult worms (eight males and eight females, separately) were placed into 4 mm cuvettes containing 100 μL of RPMI 1640 medium (Gibco, USA) and dsRNAs (Smp38.2, GFP—unspecific control and untreated) at 125 V for 20 ms. After electroporation, worms were transferred to 24-well plates with 1 mL RPMI 1640 medium (Gibco, USA) supplemented with 10% heat-inactivated Fetal Bovine Serum (Gibco, USA) and 2% Penicillin/Streptomycin (Gibco, USA). The medium was changed daily to measure relative expression using RT-qPCR during three, five, seven, and 10 days after electroporation.

### RNA Extraction, cDNA Synthesis, and RT-qPCR Analysis

All RNA extractions were performed using the TRIzol Reagent (Invitrogen, USA) method followed by the RNeasy Mini Kit (Qiagen, Germany), according to the manufacturer's guidelines. For removal of contaminant genomic DNA, samples were treated with TURBO DNA-free kit (Ambion, USA). RNAs were quantified using the Nanodrop Spectrometer ND-1000 (Thermo Fischer Scientific, USA) or the Qubit Fluorometer (Thermo Fischer Scientific, USA) and then stored at −70°C. cDNA was synthesized using the extracted RNAs and the SuperScript™ III Reverse Transcriptase (Invitrogen, USA) or the Illustra PuReTaq Ready-To-Go PCR Beads (GE Healthcare, USA), following manufacturer instructions.

Primers for RT-qPCR were strictly designed following the MIQE guidelines (23) and amplify fragments of 100–150 bp (Table S1). RT-qPCR assays were performed in three technical replicates using the Power SYBR® Green Master mix (Applied Biosystems, USA) with each primer at 200 nM in 20 mL final reaction volume in an ABI 7500 RT-PCR system (Applied Biosystems, USA). PCR efficiency for each pair of specific primers was estimated by titration analysis to be 100 ± 5% (data not shown). The specificity of the PCR product was verified by a melting curve. Internal controls to evaluate genomic DNA contaminations (RNA samples) and reagent purity (no cDNA) were included.

To assess Smp38 expression among the different stages of *S. mansoni* life cycle, absolute quantification was performed using copy number standards, i.e., 10-fold dilutions of a Smp38 clone. Copy number of each dilution was calculated through the ratio between the molecular mass of the clone and the Avogadro's constant (24). The absolute copy number of the Smp38 transcript was estimated by interpolation of the sample PCR signals from a standard curve.

In samples exposed to specific dsRNAs, Smp38 transcripts levels were analyzed by relative quantification and normalized using the *S. mansoni* cytochrome C oxidase I gene (Smp_900000). Transcript levels were analyzed using the comparative ΔCt method (25) and expressed as a percentage of difference relative to the unspecific or untreated control. Statistical analysis used the Mann-Whitney test (Wilcoxon-Sum of Ranks, *P* < 0.05). All statistical analyzes were performed using GraphPad Prism, v. 5 for Windows (GraphPad Software, La Jolla California USA, www.graphpad.com).

### Smp38-Knockdown Phenotypic Evaluation

Schistosomula were observed daily by light microscopy inversion (ABO 100–ZEISS) to check phenotypic changes and viability, such as; movement, color, tegument integrity, area, etc. On the seventh day, images of at least 100 schistosomula were recorded, and the area (μM^2^) of each schistosomulum was measured using AxioVision 4.8 software to compare the area of parasites exposed to Smp38 dsRNAs and unspecific and untreated controls. At least three biological replicates were measured. Statistical analysis employed the Mann-Whitney test (Wilcoxon-Sum of Ranks, *p* < 0.05, *N* = 3).

Adult worm motility (eight worms/1 mL medium in 24-well culture plates) was assessed for 10 days using the WormAssay software (26) to analyze parasite viability. Similarly, eight worm couples were electroporated and cultured in 6-well plates, the medium was changed daily to count the number of eggs laid.

### *In vivo* Experiments

After schistosomula exposure to dsRNAs for 4 days, 300 parasites were subcutaneously inoculated in Swiss mice (*Mus musculus*). Schistosomula treated with unspecific dsRNA-mCherry were inoculated as control. After 40 days, mice were euthanized by cervical dislocation and adult worms were recovered by perfusion (22). After perfusion, the livers from mice were removed, weighed, and treated with 10% KOH, individually, for subsequent egg counting. Egg numbers and adult worms recovered from mice inoculated with schistosomula exposed to Smp38.1 and Smp38.2 dsRNAs were compared to unspecific control group. In each experiment, we used five animals per group, and three independent biological replicates were performed. The significance of the results was tested using the Mann-Whitney (Wilcoxon-Sum of Ranks, *p* < 0.05, *N* = 3).

Additionally, worm maturation and morphological characteristics were evaluated by the injection of knockdown schistosomula in Swiss mice 4 days after dsRNA exposure (untreated, unspecific and Smp38 dsRNA treated), as previously described. After 17 and 40 days of infection, mice were euthanized by cervical dislocation and the worms were recovered by perfusion and classified according to the development classification system (Schistogram), as previously proposed elsewhere (27).

### Morphometry and Morphology of Adult Worms Recovered From Mice

The adult worms recovered after 40 days of mice infection were fixed and stored in AFA (70 and 95% alcohol, 3% formaldehyde, and 2% acid acetic), stained with 2.5% chloride carmine, dehydrated using alcohol (70, 90%, and absolute), clarified in methyl salicylate with Canadian balsam (1:2), and prepared as whole-mounts (28). Morphometric analyses were performed on male and female worms using computer images (Image Pro Plus—Media Cybernetics, USA) captured by a camera (640/480 pixels, RGB) coupled to a light microscope (Olympus BX50). The following parameters were determined: number and area of testicular lobes, ovary area, the presence of eggs and vitelline glands, the integrity of tegument, and presence and height of surface tubercles in male worms (29). Statistical significance of the morphometric data was analyzed using the Mann-Whitney test (Wilcoxon-Sum of Ranks, *p* = 0.05).

Whole-mounts were also analyzed under confocal laser scanning microscopy (CLSM), using an LSM-410, (Zeiss) equipped with a 488 nm HeNe laser and an LP 585 filter in reflected mode. We analyzed six males and six females recovered from mice infected with schistosomula previously exposed to dsRNA -Smp38, -mCherry, and untreated, in three biological replicates.

### Assessment of Egg Maturation and Viability of Miracidia

In order to evaluate the stage of maturation of eggs laid by knockdown parasites in mice infected for 40 days, 1 cm^2^ fragment of the intestine (ileum) from each mouse was removed and washed with 0.85% saline to remove feces. The tissue fragments were placed between a glass slide and a plastic coverslip and pressed with an iron press. The slides were taken under a microscope to counting and sorting the eggs (100 eggs/mouse) as previously proposed (30). The significance of the results was tested using the Mann-Whitney (Wilcoxon-Sum of Ranks, *p* < 0.05, *N* = 3).

To evaluate egg viability, Smp38 knocked-down schistosomula after 4 days of dsRNA (unspecific, Smp38.1 or Smp38.2) exposure were inoculated in Swiss mice (five mice per group). Mice were euthanized by cervical dislocation 50 days after infection and the liver of each mouse was removed. The eggs were obtained according to the technique previously described (31). The obtained eggs were then counted and used for infection of *B. glabrata* snails, lineage Barreiro de Cima. Snails with shell diameter ranging from 8 to 10 mm were used. After hatching, 200 miracidia from eggs recovered from a single mouse were exposed to 20 snails in beckers with dechlorinated water (10 miracidia/snail). The snails and miracidia were held in the light for approximately 3 h at 27°C before being placed in the aquarium. After 30 days of infection, the snail was individually exposed to light for 40 min in flasks containing dechlorinated water and examined in a stereomicroscope to verify the cercariae shedding.

### Parasite Exposure to p38 MAPK Inhibitors

SB 203580 inhibitor (Sigma-Aldrich, USA), known to inhibit the enzymatic activity of Smp38 in miracidia (13, 14), was tested in schistosomula cultures using four different concentrations (10, 25, 50, and 100 μM). Also, SB 202190 inhibitor (Sigma-Aldrich, USA), was tested in schistosomula culture in different concentrations (3, 6, 12, 25, 50, 100, 200, and 400 μM). Worms exposed to 0.02% v/v DMSO (Sigma-Aldrich, USA), only, were used as controls.

### Viability Evaluation of Smp38 Inhibited Parasites and Susceptibility to Oxidative Stress

After parasite exposure to the inhibitor, the number of viable schistosomula was quantified by staining with 5 μg/mL propidium iodide. The significance of results was evaluated by Two-way ANOVA followed by a *post hoc* Bonferroni multiple comparison test, *N* = 3.

Susceptibility of parasites depleted for Smp38 to oxidative stress was tested. First, a curve of hydrogen peroxide concentration (5 mM, 100 μM, 50 μM, 25 μM, 10 μM e 5 μM) was performed to establish a sublethal dose (data not shown). After the establishment of 50 μM of hydrogen peroxide, schistosomula treated with dsRNA-Smp38 for 4 days or exposed to SB 203580 inhibitor for 12 h were placed in 24 well-plates containing hydrogen peroxide, in triplicates. The number of dead parasites was quantified using staining with 5 μg/mL propidium iodide, 24 and 48 h after exposure. Results were analyzed by Two-way ANOVA followed by a *post hoc* Bonferroni multiple comparison test (*p* < 0.05, *N* = 3).

Expression of Glutamate-Cysteine Ligase (SmGCL, Smp_013860) and Smp38 were assessed by RT-qPCR in wild schistosomula after 5, 10, and 30 min of exposure to 100 and 200 μM of hydrogen peroxide. Also, to verify if Smp38 induces the expression of this enzyme in *S. mansoni*, we assessed SmGCL expression in Smp38 knockdown schistosomula.

### RNA Isolation, Library Construction, and High-Throughput Sequencing

To globally verify genes that expression is possibly regulated by Smp38 MAPK pathway we used an RNASeq approach. For this, total RNA from ~500,000 schistosomula exposed to 100 nM of Smp38.2 dsRNA and control for 2 days were isolated as described above. Total RNA was quantified using a Qubit fluorometer (Thermo Fisher Scientific, USA) and the quality was assessed using Agilent RNA 6000 Pico kit in a BioAnalyzer 2100 (Agilent Technologies, USA). Barcoded paired-end libraries were constructed using TruSeq® Stranded mRNA kit (Illumina, USA) according to manufacturer's instructions, using 2 μg of total RNA as input. Libraries were made equimolar, pooled with HiSeq® Rapid PE Cluster Kit v2 (Illumina), and sequenced using HiSeq® Rapid SBS Kit v2 (Illumina) on a HiSeq 2500 (Illumina) sequencer. The data was preprocessed using standard Illumina processing pipeline to segregate multiplexed reads of each sample.

### Data Processing and Differential Expression Analyses

For the sequence quality assessment, the FastQ files of each sample were submitted to FastQC (http://www.bioinformatics.babraham.ac.uk/projects/fastqc) (32). RNASeq reads are available in SRA database under accession numbers PRJNA354932 and PRJNA492452. Reads from each sample were mapped against the *S. mansoni* reference genome (v. 5.0) (33) using the STAR program (v. 2.5.0a) (34). A count table with the number of reads mapped to each transcript was obtained with the multicov sub-command of bedtools tool (v. 2.15.0). Differential expression of transcripts was performed using DESeq2 package (35) implemented in R (v. 3.3.1) (36).

The functional classification of differentially expressed genes (DEGs) in schistosomula exposed to Smp38 dsRNA was assessed using PANTHER online tool (37–39) to identify enriched Gene Ontology categories (40, 41) and KEGG pathways (42–44).

### Validation of Differentially Expressed Genes by RT-qPCR

The relative expression of a subset of DEGs between control and schistosomula treated with Smp38 dsRNA were assessed using RT-qPCR. Primers were designed as previously described to a subset of 14 genes, found as up (5) or down (7) regulated in the RNASeq dataset (Table S2). RNA extraction, cDNA synthesis, RT-qPCR reactions and analysis of transcript levels were performed as described above.

## Results

### Smp38 Expression Levels Among Developmental Stages of *S. mansoni*

**Figure 1:**
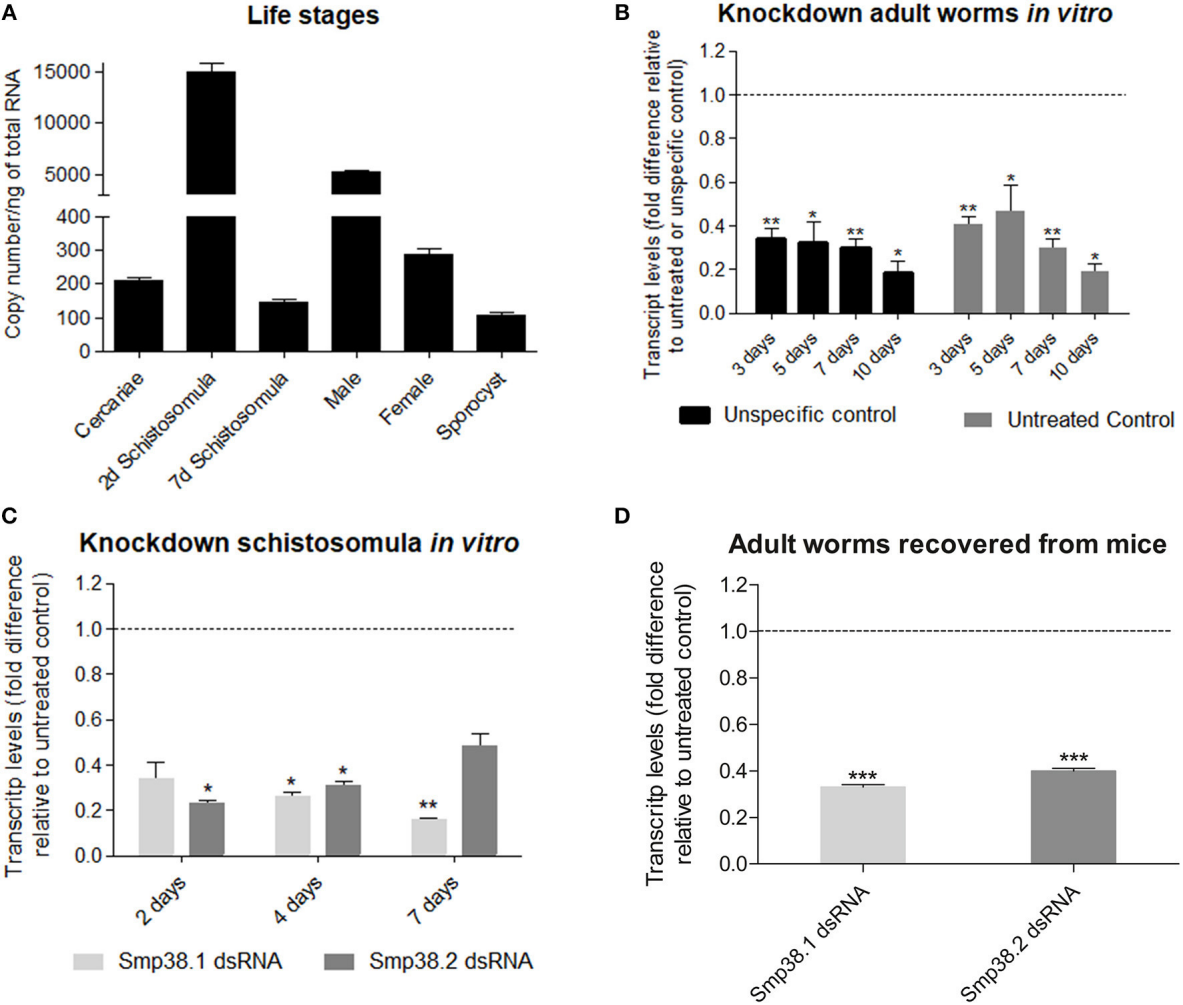
Smp38 transcript levels in the different life stages and after dsRNAs exposure of *Schistosoma mansoni in vitro* and mice recovered adult worms. **(A)** Bar graph depicting the absolute Smp38 transcript levels in the different life stages of *S. mansoni*. Absolute quantification was used to evaluate the expression levels in cercariae, 2 and 7 days schistosomula, male and female adult worms, and sporocysts are presented as copy number per ng of total RNA. Bars represent standard error of the mean of three technical replicates. **(B)** Bar graph depicting the relative Smp38 transcript levels in adult worms after 3, 5, 7, and 10 days of electroporation with Smp38.2 dsRNA. Bars represent the values relative to unspecific control (■) or untreated control (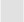) (dashed line), for each comparison, data are represented as mean fold-difference (±SE). **(C)** Bar graph depicting the relative Smp38 transcript levels in schistosomula after 2, 4, and 7 days of exposure to Smp38.1 (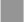) or Smp38.2 (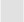) dsRNAs. For each dsRNA tested, data are represented as mean fold-difference (±SE) relative to unspecific control (dashed line). **(D)** Bar graph depicting the relative Smp38 transcript levels in *ex-vivo* adult worms exposed to Smp38.1 (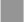) and Smp38.2 (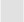) dsRNAs. Data are represented as mean fold-difference (±SE) relative to untreated control (dashed line). Transcript levels were determined by RT-qPCR and data analyzed using the ΔΔCt method and unpaired *t*-test with Welch's correction. Asterisks indicates that Smp38 transcript levels exhibit significantly difference relative to the controls; (*p < 0.05, **p < 0.005, ***p < 0.0005).

The expression profile of Smp38 in developmental stages (cercariae, two and 7 days schistosomula, adult male, adult female, and sporocyst) of *S. mansoni* was investigated by quantitative PCR. Absolute quantification was employed to normalize Smp38 expression among different developmental stages. The Smp38 gene exhibited higher expression levels in schistosomula of 2 days and male adult worms (Figure 1A).

### Suppression of Smp38 Expression in *S. mansoni* Using RNAi and Persistency of Knockdown After 40 Days

After Smp38 sequence confirmation, two different regions of Smp38 were synthesized as dsRNAs, referred to herein as Smp38.1 and Smp38.2. Smp38 transcript levels were assessed in adult worms after electroporation and in schistosomula after dsRNAs soaking.

After electroporation with Smp38.2 dsRNA, adult worms cultivated *in vitro* presented a gradual decrease in the transcript levels, reaching up to 80% reduction on the 10th day (Figure 1B).

Schistosomula treated with dsRNAs *in vitro* presented a progressive decrease in transcript levels for Smp38.1, with a significant reduction of 85% on the seventh day, while for Smp38.2, we observed a significant reduction of 78% on the second day, followed by a progressive increment in transcript levels (Figure 1C). In addition, the reduction in Smp38 transcript levels was persistent in adult worms recovered from mice after 44 days of dsRNA exposure. Smp38 knockdown was still effective with 67% reduction for Smp38.1 and 60% Smp38.2 when compared to parasites recovered from the unspecific control group (Figure 1D).

### Smp38 Knockdown Influences Parasite Survival *in vivo* but Not *in vitro*

**Figure 2:**
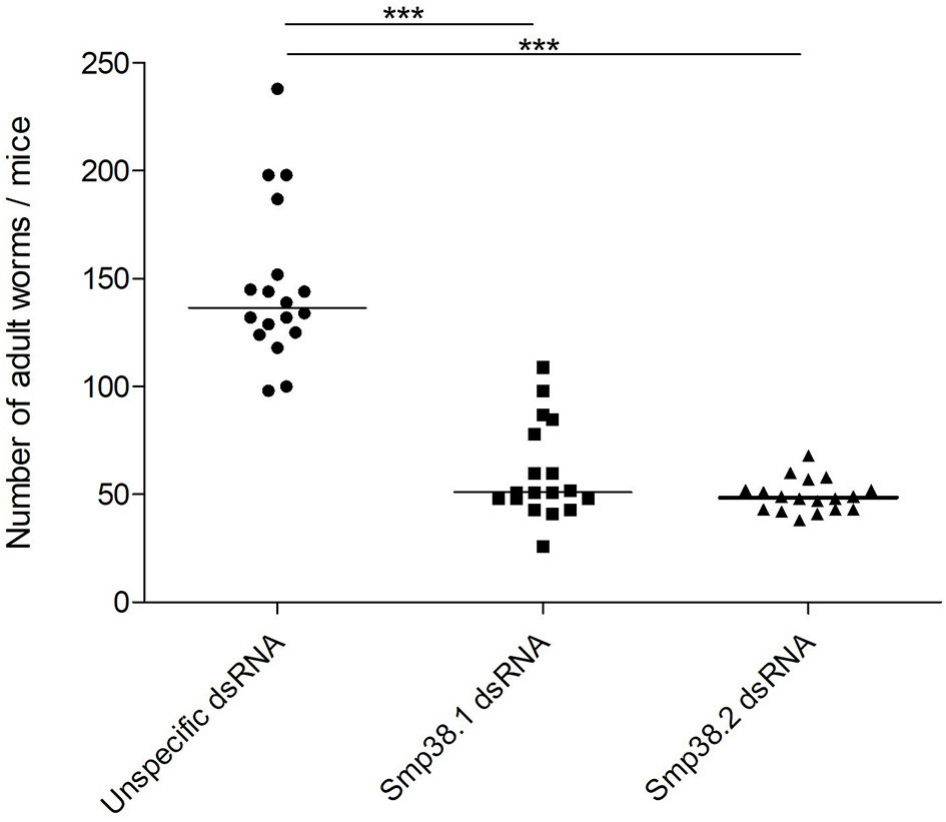
Adult worm recovery after Smp38 knockdown *in vitro* and subsequent injection of parasites into mice. Schistosomula were treated with unspecific (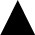), Smp38.1 (■), and Smp38.2 (▴) dsRNAs for 4 days and then injected into mice. After 40 days, mice were perfused, adult worms were recovered and counted. Each symbol in the chart represents worm counts from each mouse. The horizontal lines represent the median values per treatment group. Data were generated from 3 independent experiments and all treatments were statistically analyzed using Wilcoxon sum of ranks test. ***Significance value of *p* < 0.0001 for Smp38.1 and Smp38.2 knockdown relative to the unspecific control.

To investigate whether Smp38 knockdown influences parasite viability *in vivo*, schistosomula were incubated for 4 days with dsRNAs and then used to infect mice. The efficiency of Smp38 knockdown was checked before each infection (Figure S1). After 40 days, adult worms were perfused from the hepatic portal system and eggs recovered from the liver. Smp38 knockdown resulted in a significant decrease in the number of adult worms (46% for Smp38.1 and 67% for Smp38.2) recovered from infected mice when compared to unspecific control (Figure 2).

**Figure 3:**
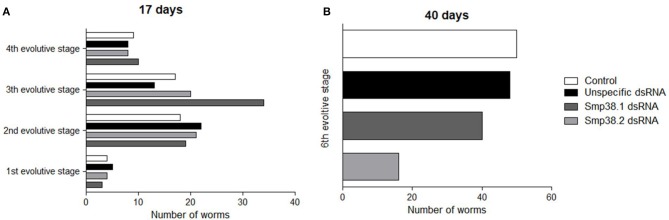
Evaluation of mice recovered adult worm maturation. Schistosomula were treated with unspecific, Sm38.1 and Smp38.2 dsRNA for 4 days *in vitro* and then injected into mice. After 17 **(A)** and 40 days **(B)**, adult worms were recovered. Horizontal bars represent the number of worms recovered from each group: control (□), unspecific control (■), Smp38.1(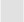), and Smp38.2 (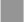) and were evaluated according to all developmental stages (1st, 2nd, 3rd, 4th, 5th, and 6th).

Then, we sought to verify the development of Smp38 knockdown parasites 17 and 40 days after mice infection. After 17 days of infection, the Schistogram (27) of all groups (Smp38 dsRNAs, unspecific, and untreated control) contained parasites from the first to the fourth stage of development (Figure 3A). Confirming previous results, after 40 days, the number of parasites recovered was reduced in the groups exposed to Smp38 dsRNA, however, all parasites were in the 6th evolutive stage of development (Figure 3B).

Females and males adult worms electroporated with Smp38.2 dsRNA and cultivated *in vitro* showed no significant differences in motility when compared to controls (data not shown).

Moreover, the area of schistosomula treated with Smp38 dsRNAs and the controls presented no significant difference (data not shown).

### Smp38 Knockdown Interferes in Adult Worms Oviposition and Egg Maturation, but Not in Miracidia Viability

**Figure 4:**
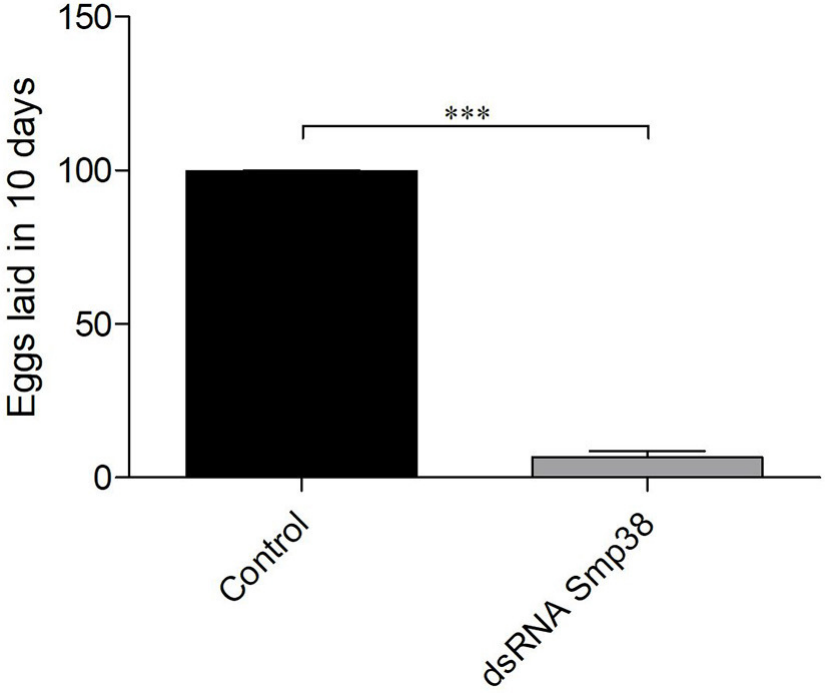
Oviposition in Smp38 knockdown adult worms *in vitro* after dsRNAs electroporation. Bar graph depicting the percentage of eggs released during 10 days after electroporation with Smp38.2 dsRNA (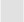) compared to the untreated control (■). Data were analyzed using unpaired *t*-test with Welch's correction (*N* = 3; ***p < 0.0001).

Adult worms electroporated with Smp38.2 dsRNA and maintained *in vitro* presented a significant reduction of 94.4% in oviposition when compared to parasites electroporated with only medium or unspecific dsRNA (Figure 4).

**Figure 5:**
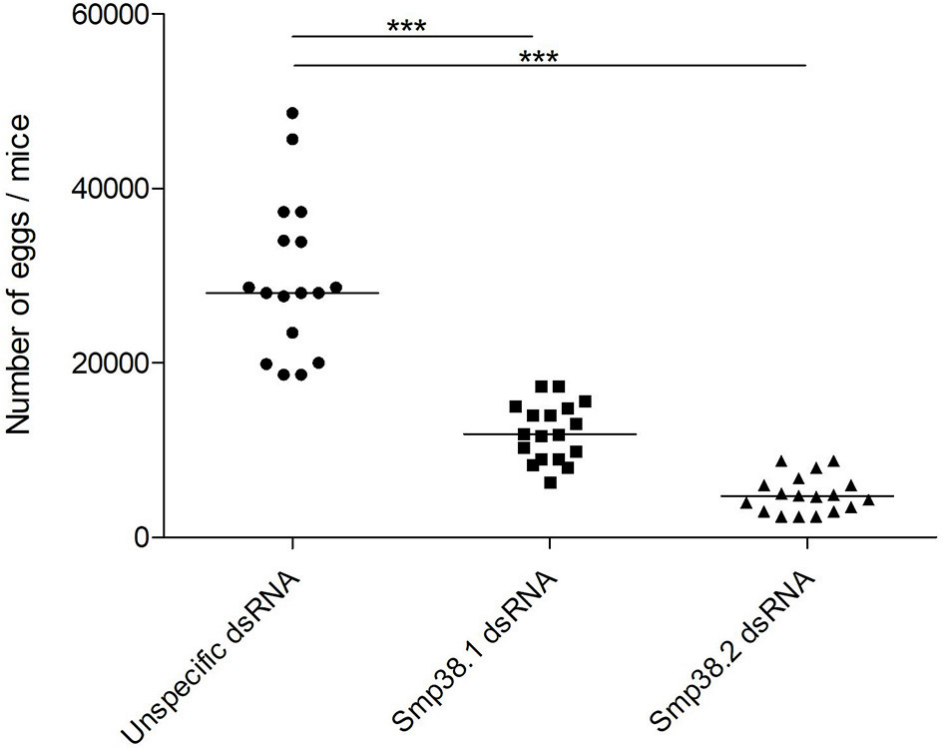
Egg recovery from liver after Smp38 knockdown *in vitro* and subsequent injection of parasites into mice. Schistosomula were treated with unspecific (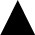), Smp38.1 (■), and Smp38.2 (▴) dsRNAs for 4 days *in vitro* and then injected into mice. After 40 days, parasite eggs per mouse liver were recovered and counted. Each symbol in the chart represents egg counts from each mouse and the horizontal lines are median values per treatment group. Data were generated from 3 independent experiments and all treatments were statistically analyzed using Wilcoxon sum of ranks test. ***Significance value of *p* < 0.0001 for Smp38.1 and Smp38.2 knockdown relative to the unspecific control.

Also, the number of eggs in the liver of mice infected with schistosomula from the different groups (Smp38.1, Smp38.2, and unspecific control) were counted and a decrease in the number of eggs was observed when compared to control group (55% for Smp38.1 and 85% for Smp38.2) (Figure 5).

**Figure 6:**
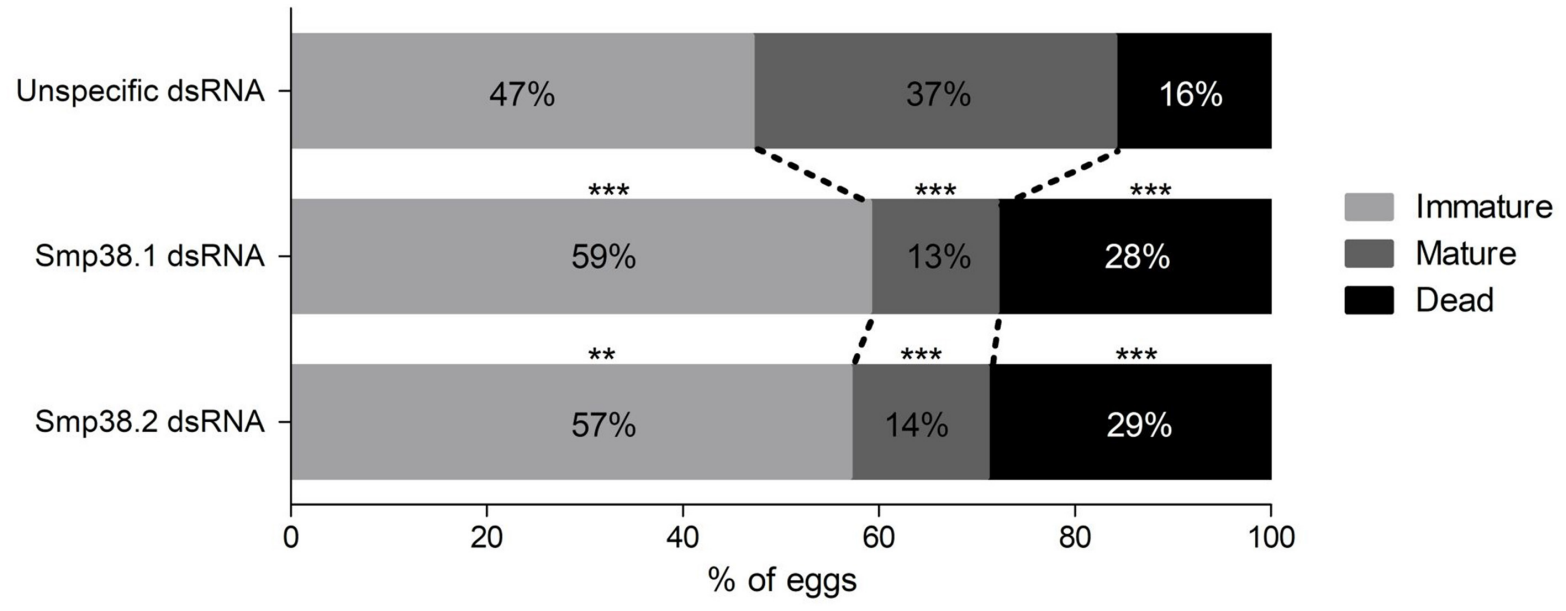
Oogram of eggs recovered from mice ileum infected with Smp38 knockdown schistosomula. The graph shows the percentage of immature (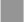), mature (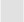), and dead (■) eggs present in the ileum intermediate portion of mice infected with Smp38 knockdown and unspecific control schistosomula. The data were treated by Wilcoxon sum of ranks test. Percentage values of each egg stage are represented inside the bars (**p ≤ 0.01, ***p ≤ 0.001), *N* = 3.

In addition, we also checked the influence of Smp38 knockdown on the egg maturation process by performing an intestinal oogram. Eggs were classified as immature, mature and dead as previously described (30). We found that ~86% of the Smp38 knockdown eggs remained in the immature stages or die before reaching the mature stage (~14%) (Figure 6).

The viability of eggs laid by Smp38 knockdown parasites was also assessed. Thus, 40 days after infection, the liver of mice infected with schistosomula exposed to Smp38 dsRNA or controls were processed. After hatching, *B. glabrata* snails were exposed to miracidia from mature eggs and, 30 days later, snails were exposed to artificial light. It was found that the same number of snails exposed to miracidia from all experimental groups was releasing cercariae. Therefore, the remaining mature eggs from Smp38 knockdown parasites were able to hatch, swim, penetrate and develop in the snail host (Figure S2). Moreover, to check the transference of the knockdown effect to a different generation, miracidia transcript level was assessed and we observed that Smp38 transcription levels of these miracidia were normalized (Figure S3).

### Smp38 Knockdown Modify Parasite Morphology

**Figure 7:**
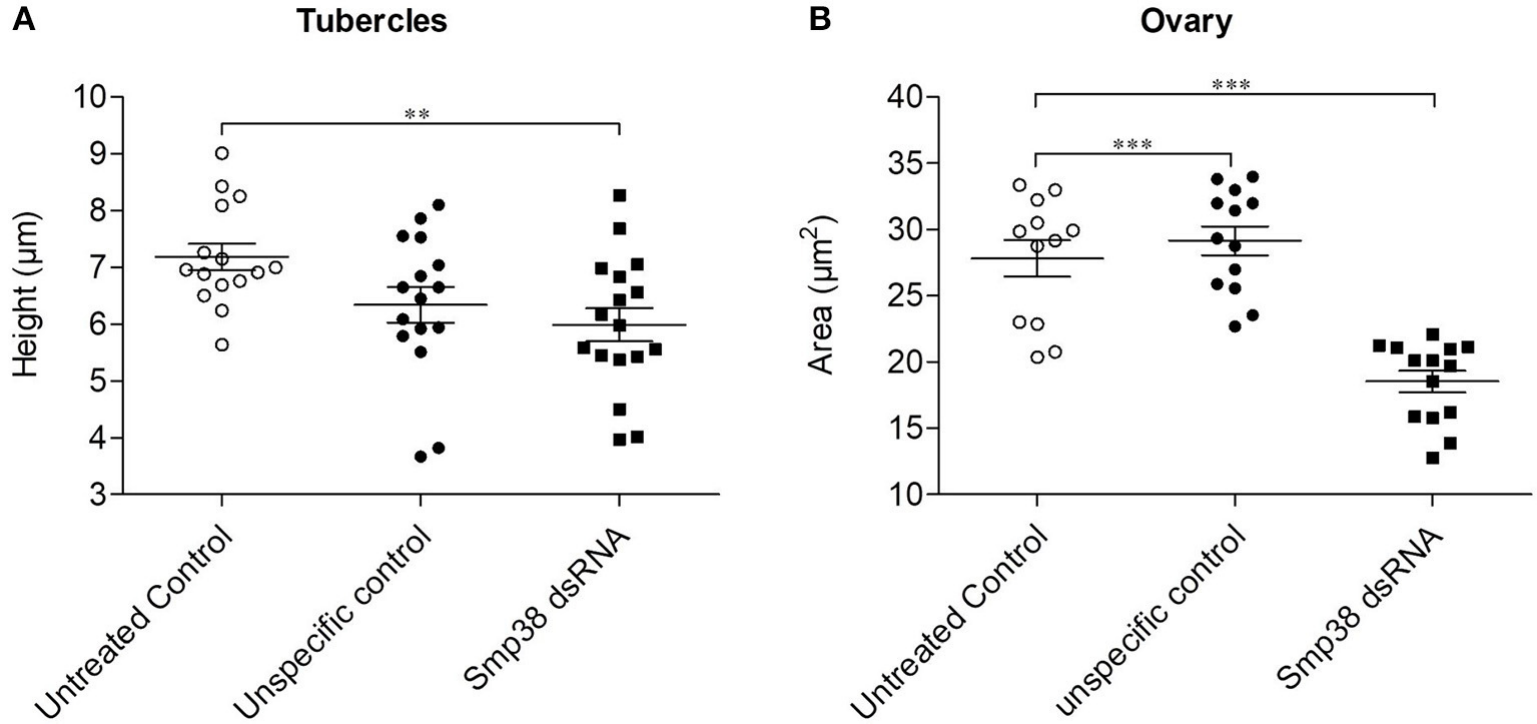
Morphometric analyses of adult worms recovered from mice infected with Smp38 knockdown schistosomula. Morphometry was performed to compare phenotypic characters of adult worms recovered after infection with Smp38 (■) knockdown schistosomula compared to worms recovered from untreated (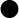) and unspecific (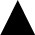) control groups. Symbols represent a single worm for each experimental group. Data were analyzed by the Mann-Whitney test. Asterisks indicates significant reduction of tubercles height **(A)** or ovary area **(B)** when compared to unspecific control and/or untreated control; (**p < 0.001, ***p < 0.0001), *N* ≥ 12.

Morphometric analyses of Smp38 knockdown adult worms showed a significant reduction (15%) in the height of the tubercles of the Smp38 knockdown males compared to the untreated controls (Figure 7A). No alterations were observed in the number and area of testicular lobes or the seminal vesicle. However, dsRNA exposed female worms showed significantly reduced ovary area (32.5%) (Figure 7B).

**Figure 8:**
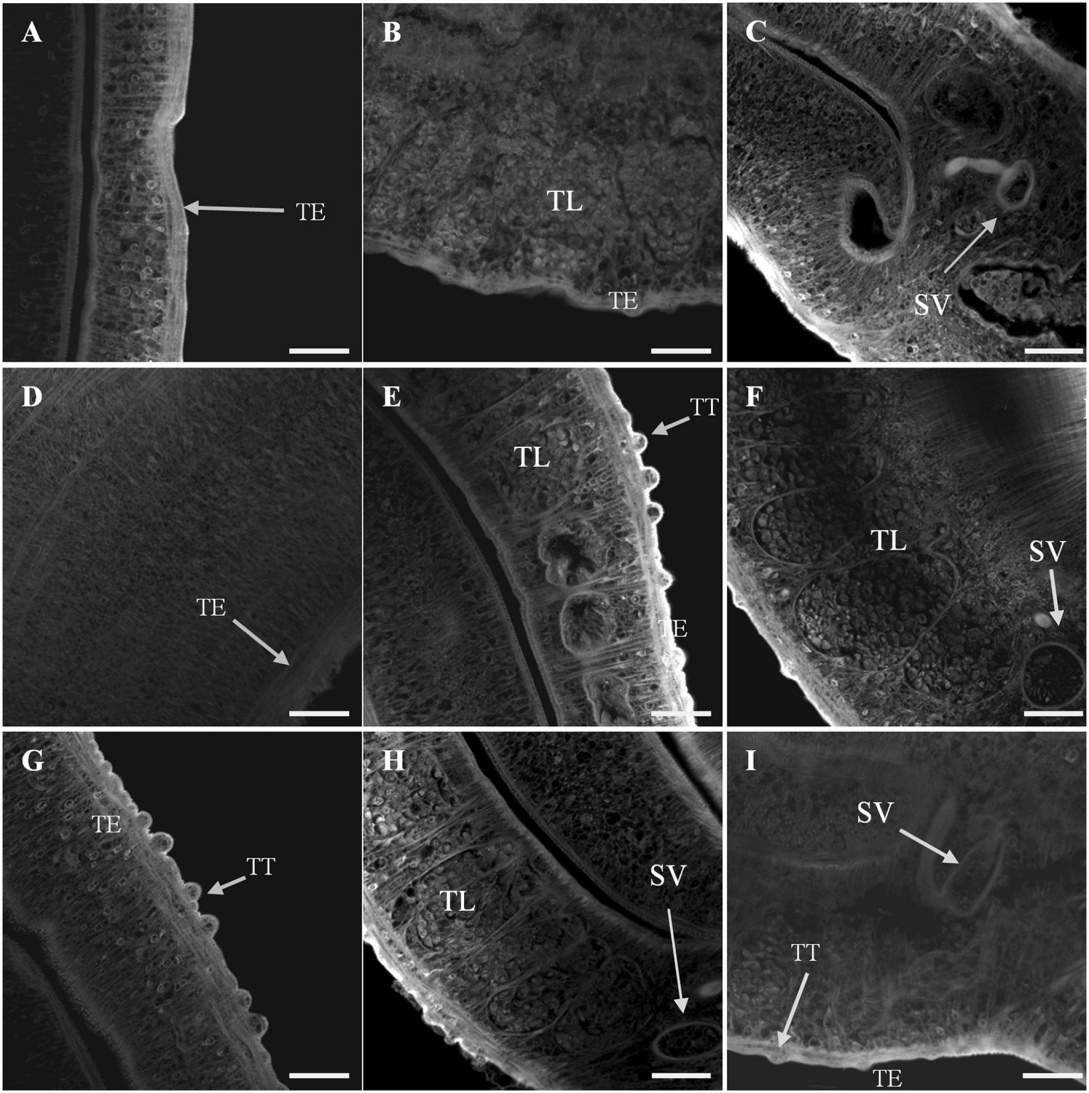
Confocal images of *S. mansoni* adult male worms recovered 44 days after Smp38 knockdown and subsequent injection of parasites into mice. **(A–D)** illustrate worms recovered from mice infected with schistosomula-treated with Smp38 dsRNA; **(E,F)** illustrate recovered worms from the unspecific control group; **(G–I)** illustrate recovered worms from the untreated control. For each experimental group, six male worms from three biological replicates were analyzed. TT, tubercles; TE, tegument; TL, testicular lobes; SV, seminal vesicle. Scale bars, 20 μm.

Confocal microscopy provided a qualitative analysis of the structural changes due to Smp38 knockdown. Smp38 knockdown worms presented morphological changes characterized by a low density of tubercles, low density, and undifferentiated germ cells within testicular lobes (Figures 8A–D). As expected, control male worms showed an intact tegument with well-developed tubercles which were regularly distributed on the parasite dorsal surface. These worms also have fully developed testicular lobes with well-differentiated germinative cells (Figures 8E–I).

**Figure 9:**
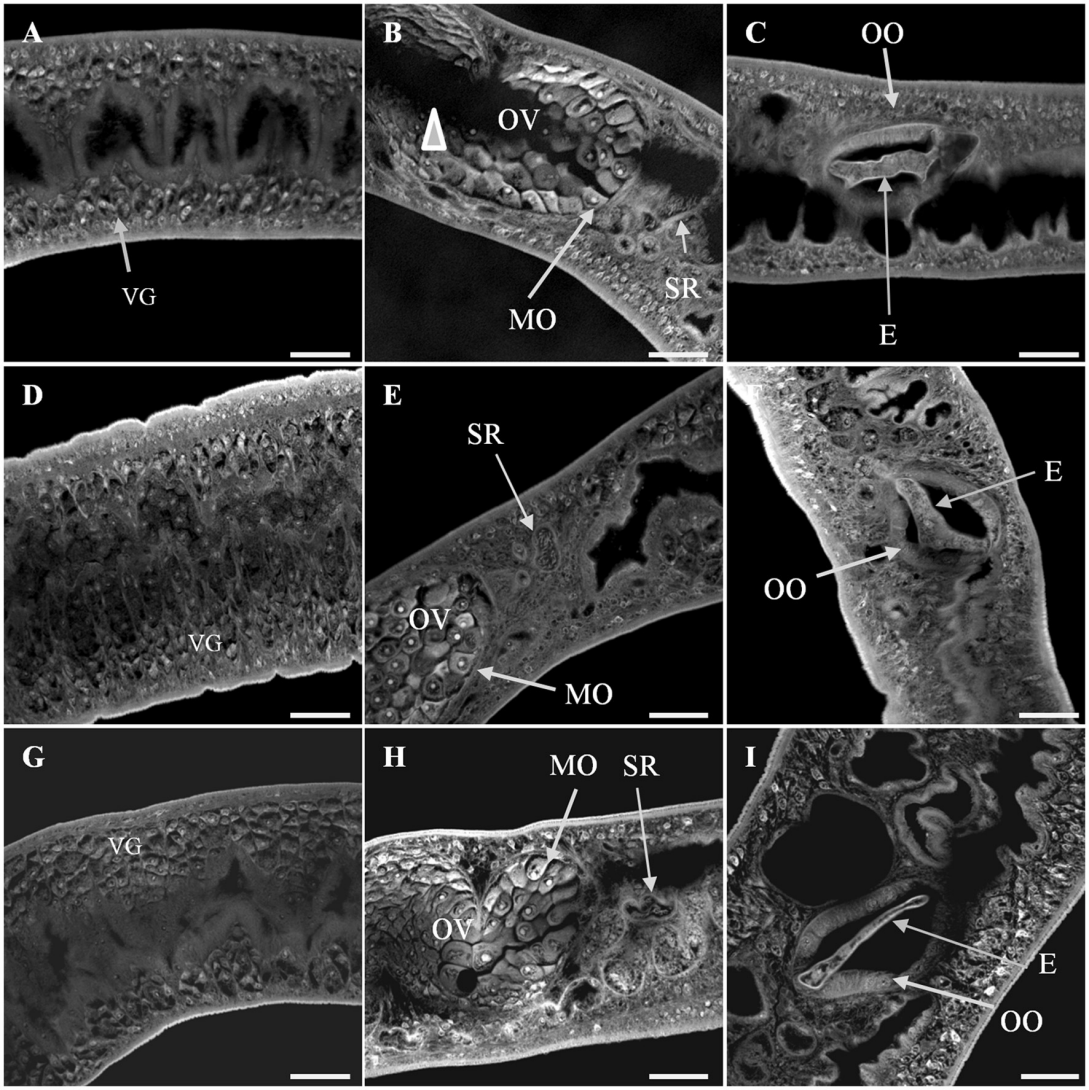
Confocal images of *S. mansoni* adult female worms recovered 44 days after Smp38 knockdown and subsequent injection of parasites into mice. **(A–C)** illustrate worms recovered from mice infected with schistosomula-treated with Smp38 dsRNA; **(D–F)** illustrate recovered worms from the unspecific control group; **(G–I)** illustrate recovered worms from the untreated control. For each experimental group, six female worms from three biological replicates were analyzed. The ovary displayed a high density of immature cells and free-cells space area (arrowhead) in **(B)**. The vitelline cells showed a lower density in the vitellarium in **(A)**. E, egg; IC, ovary immature cells; MO, mature oocytes; OV, ovary; SR, seminal receptacle; VG, vitelline glands; OO, ootype. Scale bars: 20 μm.

In addition, Smp38 knockdown females showed tegument changes characterized by profound muscular contraction. The ovary displayed a high density of immature cells, while areas free of cells and lower density of vitelline cells were observed as well. The ootype showed characteristic developing eggs (Figures 9A–D). Whereas, female worms from controls showed fully developed seminal receptacle with visible spermatozoa, vitelline glands, various stages of oocyte maturation within the ovary, including a high density of mature oocytes, developing eggs into the oocyte, and uterine eggs. No tegument abnormality was observed in the females from control groups (Figures 9E–I).

### Smp38 Biological Role Against Oxidative Stress

Later, the effect of SB 202190, a human p38 inhibitor, was evaluated in schistosomula and found not to cause alteration in schistosomula mortality in any of the eight inhibitor concentrations tested (data not shown), which could corroborate to the lack of phenotype alteration in Smp38 knockdown schistosomula *in vitro* or could be that the inhibitor is not active in the parasite protein.

**Figure 10:**
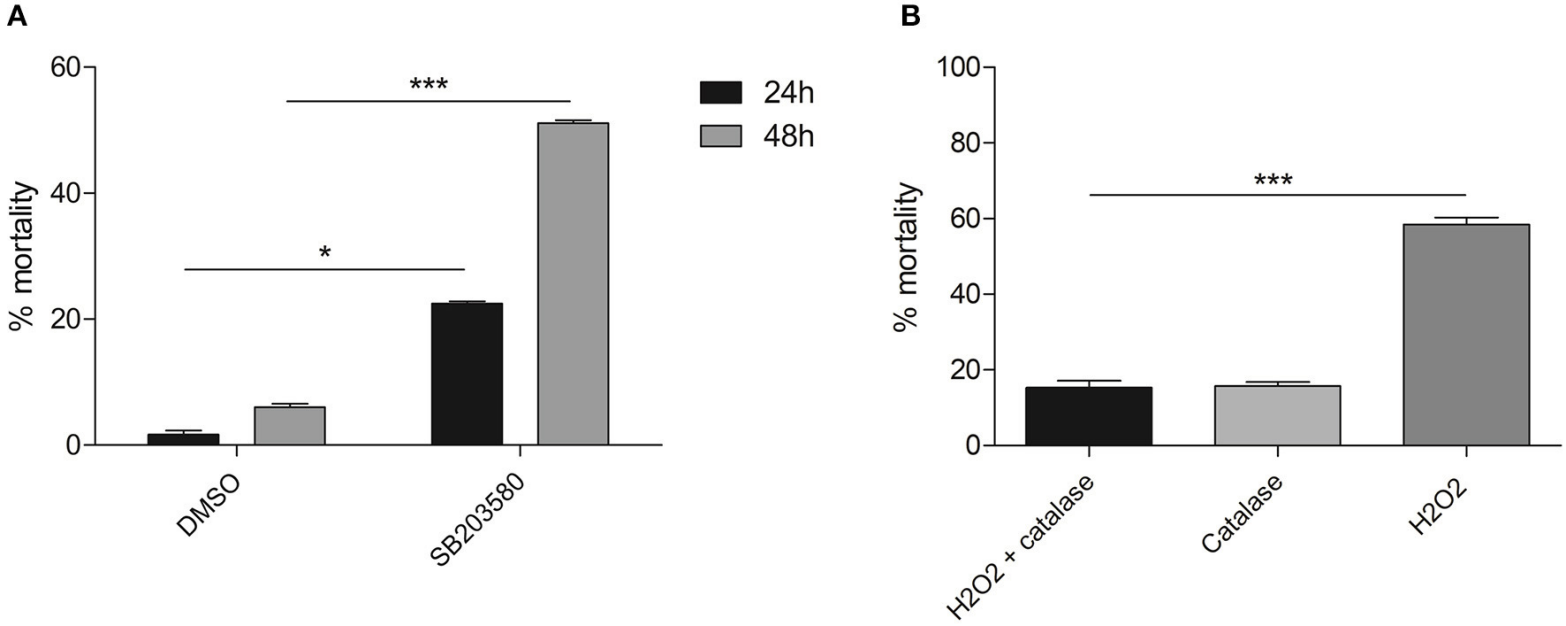
Mortality of schistosomula treated with SB 203580 inhibitor and exposed to hydrogen peroxide for 24 and 48 h. **(A)** Parasites treated with 15 μM SB 203580 for 12 h and exposed to 50 μM hydrogen peroxide. Schistosomula viability was evaluated 24 h (■) and 48 h (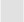) after hydrogen peroxide exposure. **(B)** Parasites treated with 15 μM SB 203580 for 12 h and exposed to 0.025% bovine catalase only (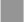); 50 μM hydrogen peroxide in the presence (■) or absence (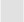) of bovine catalase. Schistosomula viability was evaluated 24 h after hydrogen peroxide exposure. Significance was analyzed by Two-way ANOVA and significant results were treated by the Bonferroni's test (*p < 0.05, ***p < 0.001, *N* = 3). Bars represent the standard error between replicates.

Another inhibitor, SB 203580, has been demonstrated to attenuate Smp38 phosphorylation activity in a dose-dependent manner (13, 14). To investigate the potential role of Smp38 in parasite evasion from oxidative stress, first we performed a viability curve using six concentrations of hydrogen peroxide and defined 50 μM of hydrogen peroxide as a sublethal dose (data not shown). Subsequently, schistosomula were exposed to four different SB 203580 concentrations (Figure S4) and 15 μM was the dose in which schistosomula were viable for at least seven days after SB 203580 inhibitor exposure (data not shown). After the establishment of a sublethal dose for both, hydrogen peroxide and inhibitor, schistosomula were exposed to 15 μM of SB 203580 for 12 h and then exposed to 50 μM hydrogen peroxide. Schistosomula treated with the SB 203580 inhibitor were considerably more susceptible to oxidative stress after 24 and 48 h of hydrogen peroxide exposure, showing an increase in mortality of 21.6 and 45%, respectively, when compared to control parasites (0.02% DMSO) (Figure 10A). To ensure that hydrogen peroxide was the oxidant factor, 12 h after exposure to SB203580 inhibitor, parasites were exposed to 50 μM H_2_O_2_ in the presence or absence of 0.025% bovine catalase (neutralizing agent), and the viability was assessed after 24 h (Figure 10B). In the presence of catalase, no decrease in viability was detected, while, again, in the presence of hydrogen peroxide the same levels were detected.

**Figure 11:**
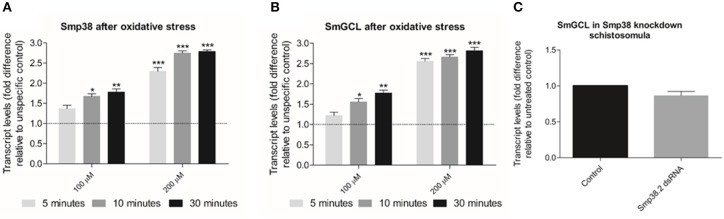
Evaluation of Smp38 and SmGCL transcript levels in Smp38 knockdown schistosomula and after parasite exposure to exogenous oxidative stress. Bar graph indicating the relative steady-state transcript levels of Smp38 **(A)** or SmGCL **(B)** 5 (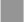), 10 (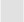), and 30 min (■) after exposure to 100 and 200 μM of hydrogen peroxide relative to transcripts levels in schistosomula not exposed to oxidative stress (control). Data are represented as mean fold-difference (±SE) relative to unspecific control (dashed line). **(C)** SmGCL transcript levels in schistosomula from untreated control (■) or exposed to Smp38.2 dsRNA (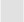). Transcript levels were determined by RT-qPCR and data analyzed using the ΔΔCt method (25), followed by statistical analysis using the Mann-Whitney test (Wilcoxon sum of ranks) (*p < 0.05, **p < 0.01, and ***p < 0.001), *N* = 3. Bars represent the standard error between replicates.

Expression of Glutamate-Cysteine Ligase (SmGCL) in *B. malayi* and *C. elegans* is induced by the Smp38 MAPK signaling pathway (45, 46) and its expression is triggered by oxidative stress. To verify if Smp38 also induces the expression of this enzyme in *S. mansoni*, we assessed Smp38 and SmGCL transcripts levels in wild schistosomula exposed to hydrogen peroxide and Smp38 knockdown schistosomula. Smp38 and SmGCL transcript levels in schistosomula increases after oxidative stress (Figures 11A,B). However, SmGCL transcript levels are not reduced in Smp38 knockdown schistosomula (Figure 11C).

### Transcriptome Profiling in Smp38 Knockdown Schistosomula

To examine the *in vitro* effect of Smp38 knockdown in schistosomula and aiming at identifying genes regulated by the Smp38 MAPK pathway we analyzed the knockdown parasite transcriptome. Schistosomula were exposed to Smp38 dsRNA, and after 48 h of incubation, RNA was extracted, and the total transcriptome analyzed. The efficiency of Smp38 MAPK knockdown at this time point was assessed using RT-qPCR (Figure S5). We generated 4 paired-end libraries yielding a total of 56.8 Gb data, with more than 9 Gb of data per sample (Table S3). Biological replicates showed a good correlation with samples grouped according to the treatment condition (Figure S6A).

To verify differentially expressed genes (DEGs) after Smp38 dsRNA exposure, we compared expression profiles of Smp38 depleted schistosomula with the untreated control. RNASeq analysis resulted in 1,154 DEGs (766 down-regulated and 388 up-regulated) (padj < 0.01). A complete list of DEGs detected is provided in Table S4. The magnitude distribution for the differentially expressed genes is illustrated using an MA plot analysis (Figure S6B).

Twelve DEGs from the Smp38 knockdown and untreated control samples were selected to validate the RNASeq results. Their relative expression levels were determined by RT-qPCR. Results showed a concordance between RNASeq and RT-qPCR data (Figure S7).

### Functional Analysis of Gene Categories Altered in Smp38 Knockdown Schistosomula

**Figure 12:**
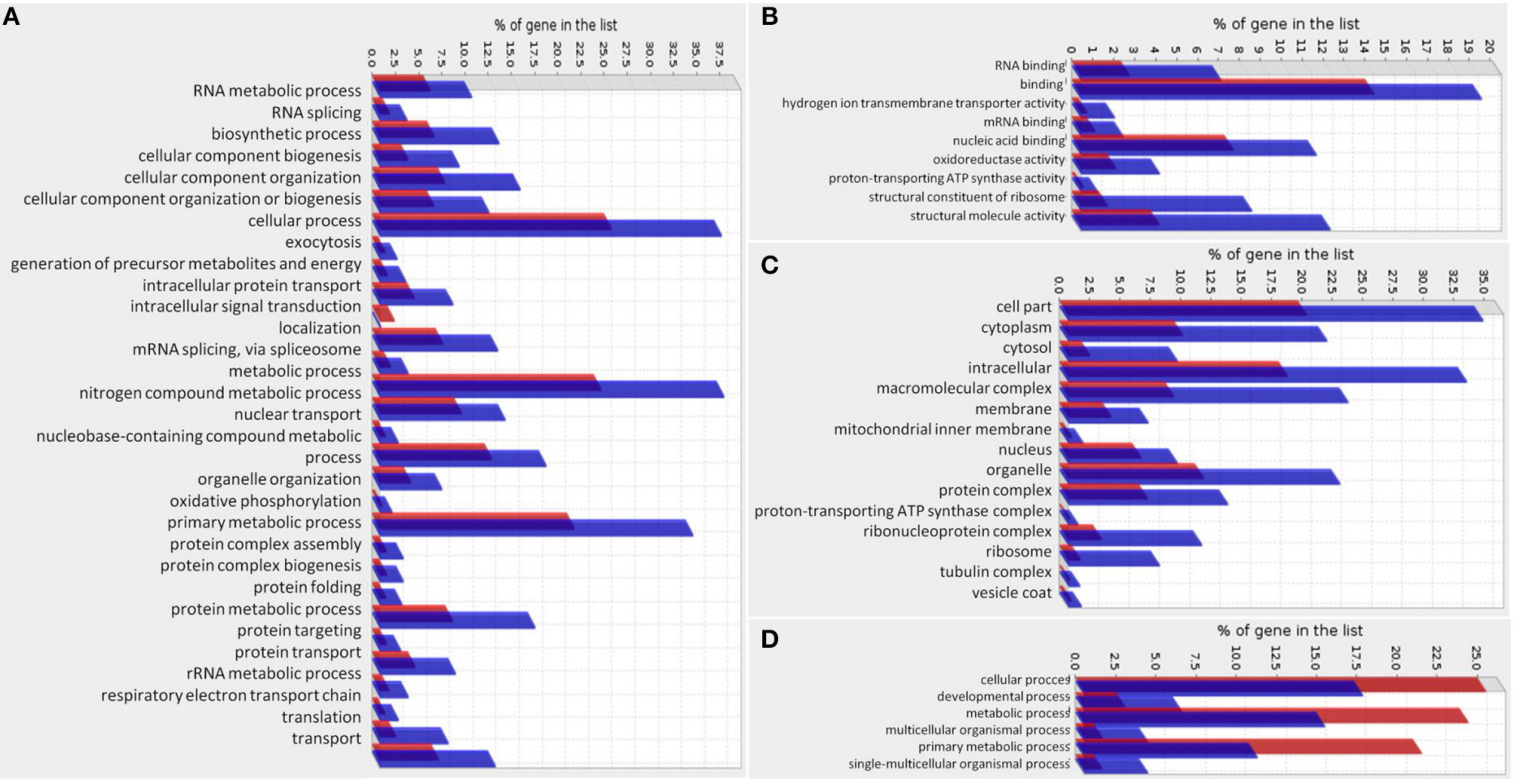
Gene Ontology enrichment of genes regulated by Smp38 MAPK pathway. Gene Ontology enrichment of significantly down-regulated genes in Smp38 knockdown schistosomula is depicted separately into three categories: **(A)** Biological Process, **(B)** Molecular Function, and **(C)** Cellular Component. Gene Ontology enrichment of significantly up-regulated genes in Smp38 knockdown schistosomula presented only the Biological Process category with enriched subcategories **(D)**. In red are represented the proportion of genes in the predicted proteome of *S. mansoni* for each subcategory; in blue, the proportion of DEGs in each subcategory.

We analyzed the gene function of DEGs and compared the functions to the total *S. mansoni* protein-coding genes. For down-regulated genes, we found that the biological processes enriched categories in Smp38 dsRNA treated parasites are related to diverse cellular processes, among those, we highlight: oxidative phosphorylation, translation, protein folding, nuclear transport, and nitrogen compound metabolic process (Figures 12A–C). For up-regulated genes in Smp38 knockdown schistosomula, only the Biological Process category presented enriched subcategories: cellular, developmental, metabolic, multicellular organismal, primary metabolic, and single-multicellular organismal processes (Figure 12D).

## Discussion

Many extracellular stimuli are converted into specific cellular responses through the activation of mitogen-activated protein kinases (MAPKs). The p38 MAPK signaling cascade is preferentially activated by non-mitogenic stimuli, such as environmental stress and proinflammatory cytokines (8).

In the present report, we demonstrated that Smp38 MAPK is involved in parasite survival and reproduction when exposed to the host immune system, as there was a significantly lower number of adult worms and eggs recovery from Smp38 knockdown schistosomula. Despite all parasites recovered from mice infected with Smp38 knockdown schistosomula were in the 6th evolutive stage after 40 days, Smp38 is likely related to parasite survival and maturation of reproductive organs.

In addition, our results demonstrate that Smp38 knockdown causes phenotypic changes in the reproductive biology of schistosomes. It has been long known that paired worms migrate to mesenteric venules (47), where females lay hundreds of eggs daily. It is noteworthy that females cannot undergo dislocation on their own because they lack a specialized tegument and musculature, which is present in male worms (48). One possible implication is that migration could be hampered since Smp38 knockdown males presented lower tubercles height which helps in the adherence to the walls of the blood vessels, which could influence female oviposition in the mesenteric veins.

Over the last years, several studies focused on investigating gene function in *S. mansoni* evolutive stages using RNAi (49–51). It has even been shown that some genes, when knocked down at an early stage of parasite development, such as schistosomula, may be sufficient to promote long-term changes that affect the growth and development of the worm in the mammalian host (49, 52). In the present study, confocal images markedly showed that Smp38 knockdown in schistosomula induced changes in the reproductive organs of adult worms from both genders. In males, despite the changes in the structural organization of testicular lobes, this fact does not seem to have affected spermatogenesis. The finding of spermatozoa within the seminal vesicle in male worms and stored in the seminal receptacle of females provide support for this hypothesis.

A recent study showed differences in gene expression between the anterior (ootype) and posterior end (vitellarium, ovary, and seminal receptacle) of female adult worms (53). Consistent with previous investigation of the roles of SmERK1 and SmERK2 (49), our results showed that the vitellarium and the ovary were target organs in parasites knockdown for those MAPKs, which could impair eggs production. After Smp38 knockdown, females showed vitellarium with reduced density of vitellocytes, cells that play a key role in egg production by providing nutritive reserves (lipid droplets and glycogen particles) for the developing embryo into the ootype. Additionally, the ovary was disturbed with a high density of immature oocytes, egg production was dramatically reduced, and oocytes were not tightly packed. It remains possible that Smp38 knockdown impaired the physiological process of vitellocyte and oocyte production, as well as, oocyte maturation (54).

Although the male and female reproductive systems of Smp38 knocked-down parasites showed relevant morphological changes, in females, developing eggs within the ootype were observed. These findings raise some questions. In the absence of Smp38 are the eggs produced viable? Are the larvae able to infect the intermediate host?

In seeking answers to some of these questions, we observed that most of the eggs of the Smp38 knockdown females remain in the immature stages or die before reaching maturity. Smp38 is therefore essential for *S. mansoni* egg maturation and a potential target for drug development. We found that Smp38 knockdown is persistent in adult worms, interfering with egg maturation, but transcription levels were recovered in miracidia, not interfering with miracidia infectivity and development in the snail host. The involvement of Smp38 in miracidia ciliary movement and larvae development in the snail host has been previously demonstrated (13, 14), but, due to the normalization of Smp38 transcript levels in miracidia, no alteration in the phenotype was observed. It is worth noting that mature eggs may have been produced by females refractory to RNAi treatment since heterogeneity of dsRNA uptake in larvae has been demonstrated (55).

During *S. mansoni* infection, cells from hosts defense system respond to the presence of parasites and eggs producing reactive oxygen species (ROS), such as superoxide radical (O${{}^{2}}_{.-}$) and hydrogen peroxide (H_2_O_2_), generating oxidative stress (56). Therefore, ROS has been shown to play a crucial role in the host defense mechanism against parasites (57). Parasites have evolved strategies to evade the immune system, one such mechanism is the expression of antioxidant enzymes, which are essential for protection against ROS (19, 50). The role of p38 MAPK in the activation of protection responses against oxidative stress has already been studied in several organisms (45, 46, 58) but remains unknown in *S. mansoni*.

Schistosomula treated with the SB 203580 inhibitor were significantly more susceptible to oxidative stress, indicating that the parasite activates the p38 MAPK signaling pathway in reaction to ROS exposure. Additionally, we have shown that oxidative stress in schistosomula increases Smp38 and SmGCL transcription levels. However, SmGCL expression does not seem to be regulated by Smp38 pathway as in other organisms like *C. elegans* and *B. malayi* (45, 46). Yet, the parasite seems to require a robust p38 MAPK pathway activation for the detoxification of ROS, once Smp38 knockdown *S. mansoni* seem to regulate many other enzymes related to oxidative stress response, such as Thioredoxin 1 (Trx1, Smp_008070), Glutathione peroxidase (Smp058690), Glutathione-S-transferase (GST-26, Smp_102070), Methionine sulfoxide reductase (Smp_196790), Lactoylglutathione lyase (GLX I, Smp_001410) (50, 59–61).

To elucidate which genes are modulated by the Smp38 MAPK in the schistosomula life stage, RNASeq was used to globally evaluate the effect of Smp38 knockdown. We observed that there was an enrichment of genes included in the category of structural composition of ribosome and polypeptides synthesis; those were down-regulated (S8 Figure S8) in samples treated with Smp38 dsRNA. Under normal conditions, ribosomal proteins and RNAs are synthesized in stoichiometric amounts (62). The differential expression of specific ribosomal proteins has been reported in several pathological conditions, could be a signal of a systemic disruption of physiological regulatory mechanisms (63), or be involved in the adaptation to stress, as has been reported for the tsetse fly during stress caused by trypanosome invasion (64).

We also observed a significant decrease in the expression of genes with structural functions, such as those encoding the tubulin and collagen type 1 proteins. Accordingly, inhibition of the p38 MAPK and Smad2/3 signaling pathways has been shown to reduce collagen expression type 1 in hepatic cells and the inhibition of both pathways resulted in a complete blockage of collagen type 1 expression (65). Inhibition of p38 MAPK activation in human epithelial cells also down-regulated the expression and synthesis of type 1 collagen induced by TGFβ-2 (66). Since schistosomula develop a heptalaminate tegument membrane composed by different proteins and glycoproteins (tubulin, collagen) (67), decreased expression of those proteins could contribute to a generalized disorganization of tissues earlier on in the parasite development, thus reducing their protection against the host's immune system, reducing survival.

Further, Smp38 knockdown parasites exhibited several down-regulated genes related to spliceosome; those were highlighted in the KEGG pathway (Figure S9) and are likely another example of a systemic effect on schistosomula homeostasis. Splicing factor expression reflects the specific patterns of alternative splicing in different cells and tissues (68). It has already been demonstrated that p38 MAPK can control tumor suppressor PTEN expression and cytokine production through U6atac modulation, a minor spliceosome snRNP (69).

The oxidative phosphorylation pathway also presented significant enrichment in down-regulated genes in knocked-down parasites (Figure S10). p38 MAPK may play an important role in controlling the biogenesis of mitochondrial proteins (70), being activated after muscle contraction in rodents and humans (71). Furthermore, the mitochondrial oxidative phosphorylation system plays a fundamental role in the production of energy, in the generation of free radicals, and in apoptosis (72). Unbalance of these proteins can alter the electron transport chain functioning and the maintenance of the mitochondrial membrane potential, leading to an accumulation of free radicals and triggering a higher susceptibility to oxidative stress. We also observed a decrease in the expression of genes encoding vacuolar H^+^-ATPases (v-ATPases). The decrease in v-ATPase expression is related to a greater sensitivity to oxidative stress by H_2_O_2_ in yeast (72). Such knockdown effects could severely compromise schistosomula development in the host.

Genes related to purine (Figure S11) and pyrimidine metabolism also showed decreased expression as a result of Smp38 MAPK knockdown. This fact is very relevant since *Schistosoma* does not use the *de novo* pathway for purine nucleotide synthesis (73). The pathways involved in purine metabolism are highly regulated and is a choke point for parasite survival (74).

Also, the differential expression of genes related to immune responses, especially those mapped in the following pathways: antigen presentation by MHC class I, HMGB1-RAGE inflammatory, and IL-3 signaling via ERK and PI3K were verified. It is known that the parasite modulates the host immune response through the mimicry of host molecules on its surface (75). Thus, changes in the expression of these genes may alter the way the parasite manages to escape and thus migrate and survive within the host.

No remarkable GO enrichment was observed for the up-regulated genes. Although many of the DEGs (up- and down-regulated) are found in one (or more) GO functional categories, we observed that approximately 27% of DEGs are annotated as hypothetical or uncharacterized proteins, for which there is no GO annotation available. These uncharacterized proteins may be related to or responsible for some of the phenotypic alterations observed in this study, may represent parasite-specific genes, and promising targets in schistosomula for further investigation.

In summary, the data presented here demonstrate, for the first time, the importance of Smp38 in the schistosomula development into adult worms in face of the host immune system, since fewer worms were recovered when Smp38 was knocked-down and these worms presented morphological changes as a low density of tubercles. Smp38 knockdown also affects the parasite reproduction, demonstrated by the malformation of reproductive structures in females, decreased oviposition *in vitro* and *in vivo*, and a significant increase in the number of immature and dead eggs *in vivo*. Smp38 MAPK pathway is proven to be essential for parasite protection against endogenous and exogenous oxidative stress sources, as was shown *in vivo* and by the regulation of expression of several genes related to detoxification mechanisms. Moreover, the functional characterization of the targets regulated by Smp38 MAPK may reveal new strategies to combat schistosome parasites, besides the contribution to a better understanding of the parasite development and interaction with the definitive host.

## Author Contributions

SG, LA, GO, and MM contributed conception and design of the study. SG, LA, MS, NA, NT, AK, and AM performed the experiments. LA, RN, and JM-S obtained and analyzed confocal data. SG performed the bioinformatic analysis. SG, LA, and MM performed statistical analysis. GO and MM contributed with reagents, materials, analysis tools. SG, LA, RN, JM-S, GO, and MM wrote the manuscript. All authors contributed to manuscript revision, read, and approved the submitted version.

### Conflict of Interest Statement

The authors declare that the research was conducted in the absence of any commercial or financial relationships that could be construed as a potential conflict of interest.
