# Supplementary figures and images for: Smp38 MAP Kinase Regulation in Schistosoma mansoni: Roles in Survival, Oviposition, and Protection Against Oxidative Stress
Source: Front Immunol. 2019 Jan 24;10:21. doi: 10.3389/fimmu.2019.00021 (PMC6353789; doi:10.3389/fimmu.2019.00021)

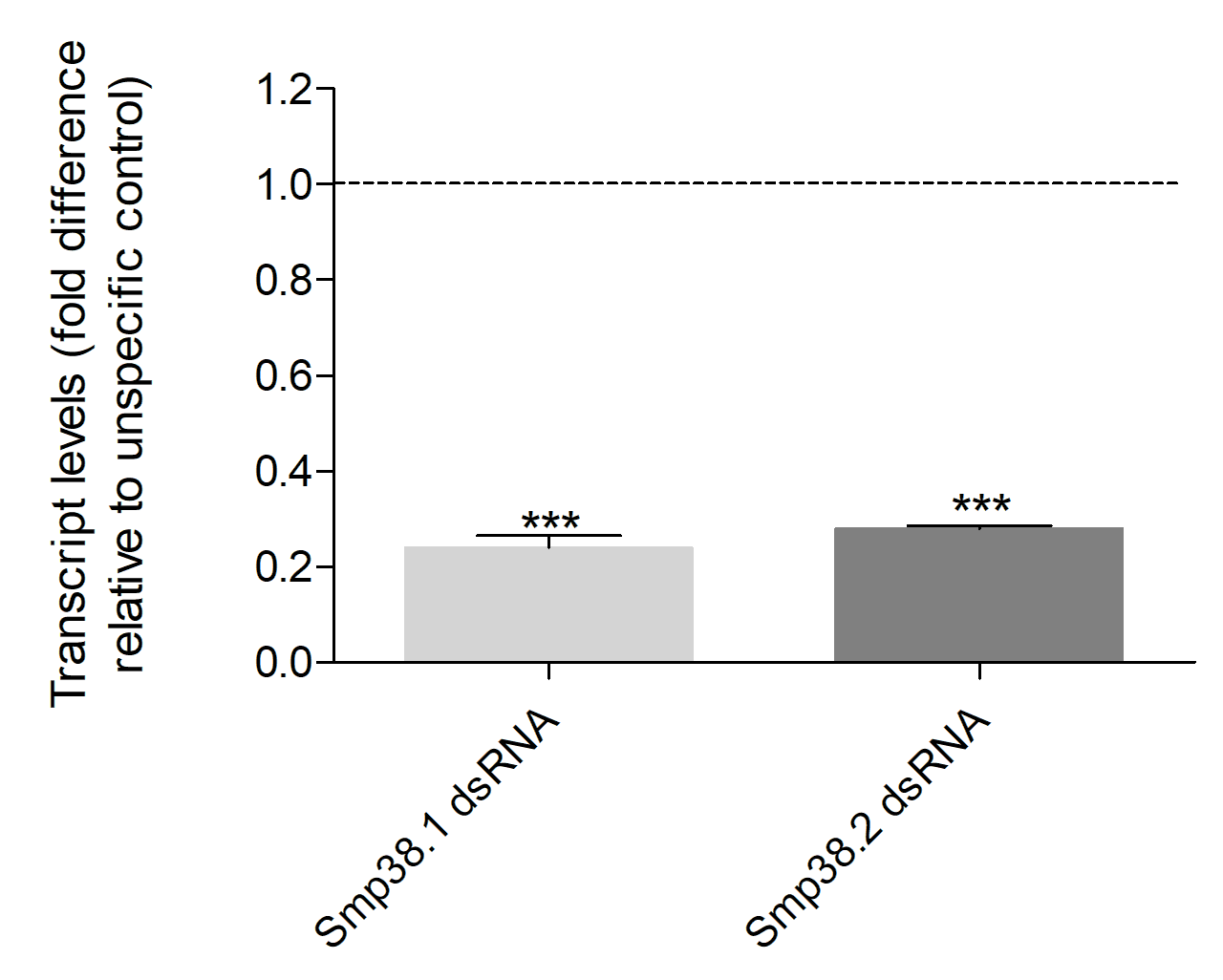

Supplement: Supplementary file 1 [file Image_1.TIF]

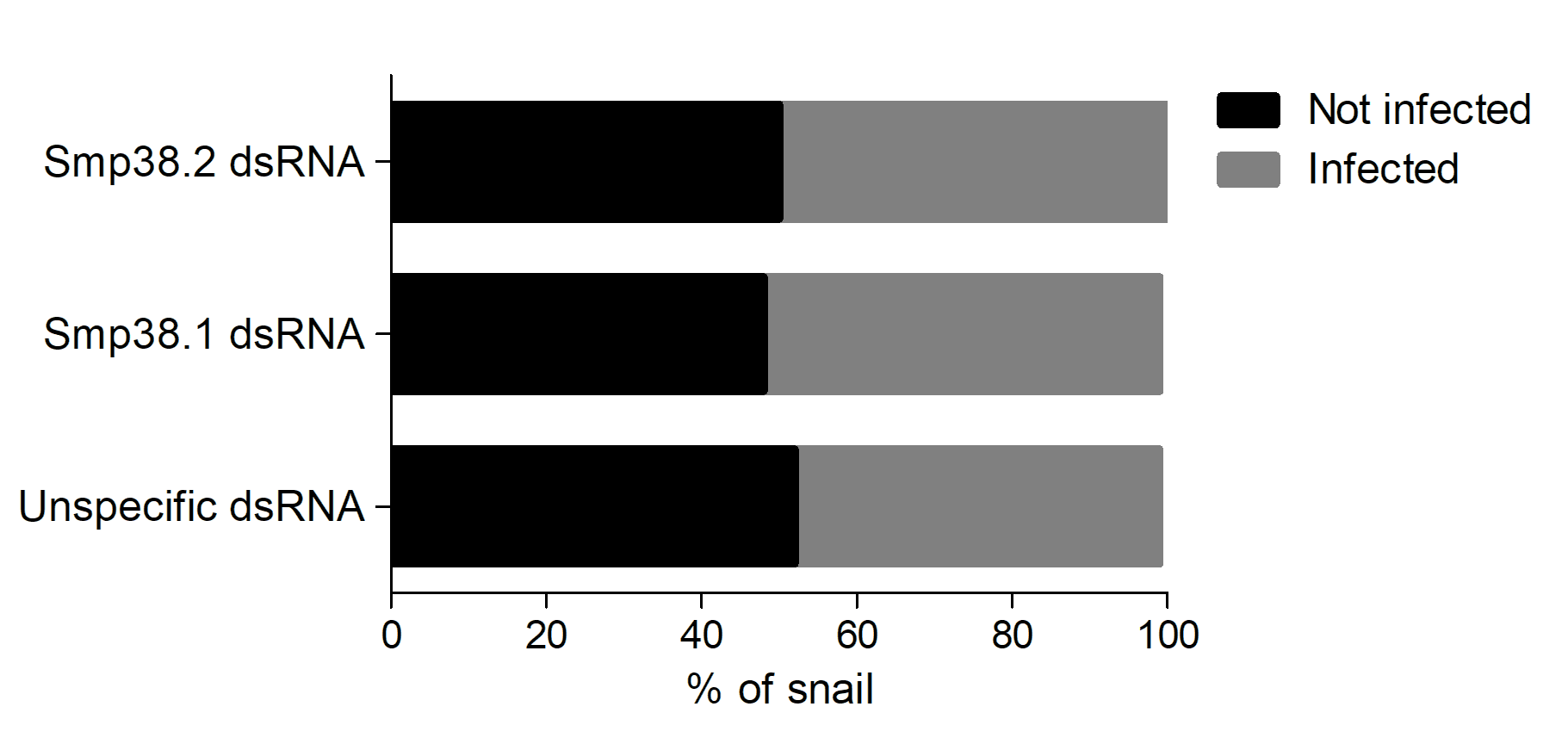

Supplement: Supplementary file 2 [file Image_2.TIF]

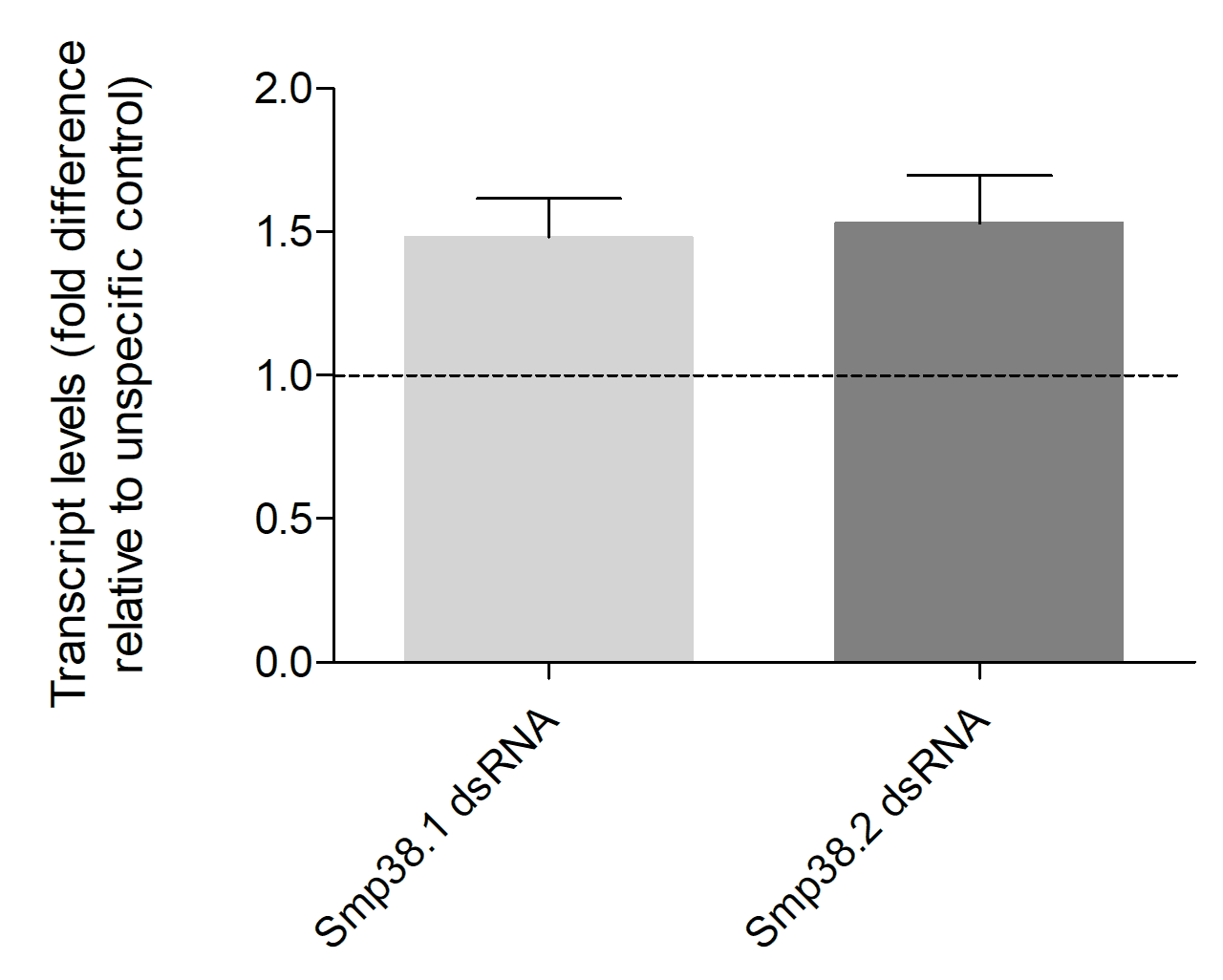

Supplement: Supplementary file 3 [file Image_3.TIF]

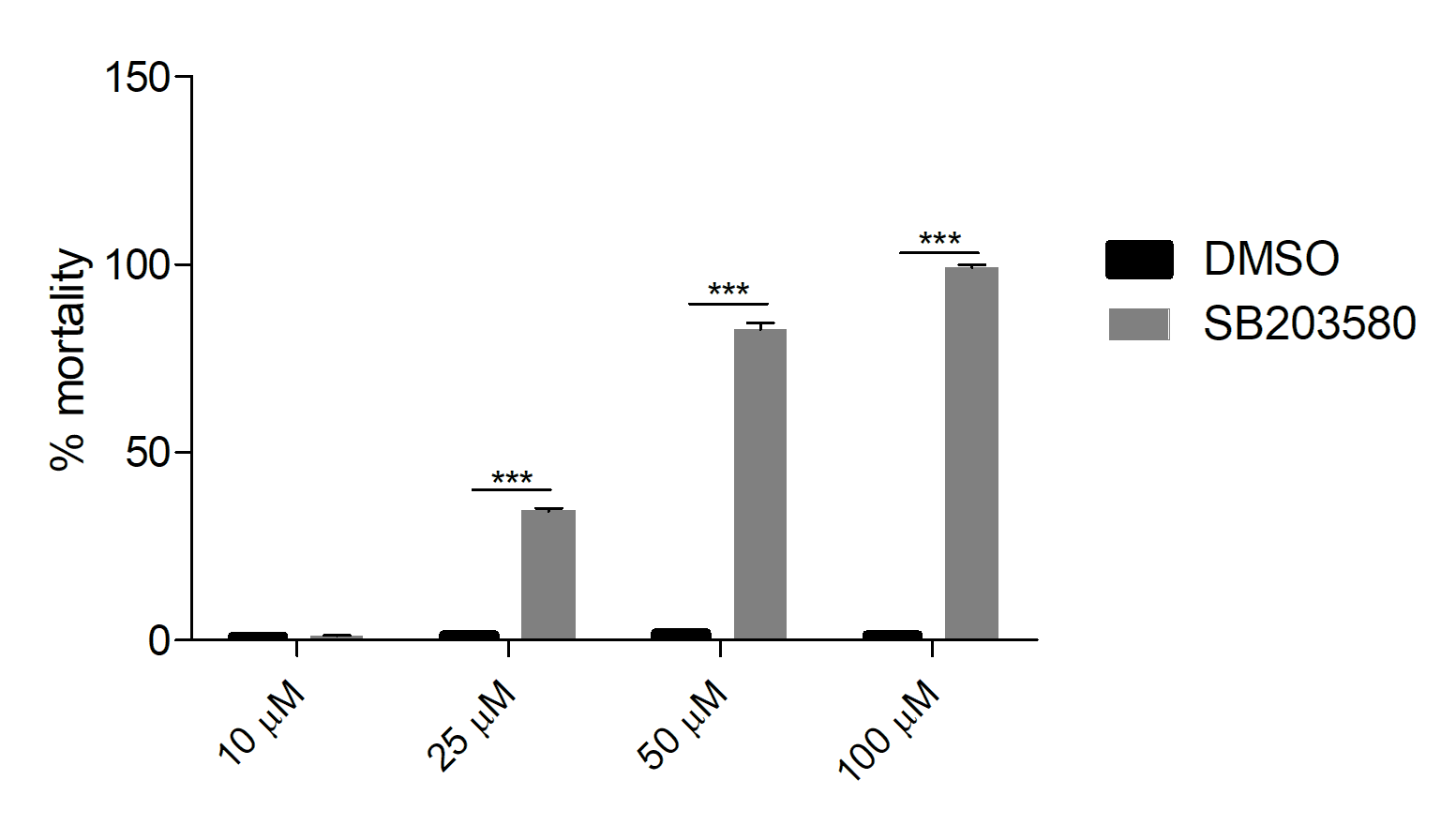

Supplement: Supplementary file 4 [file Image_4.TIF]

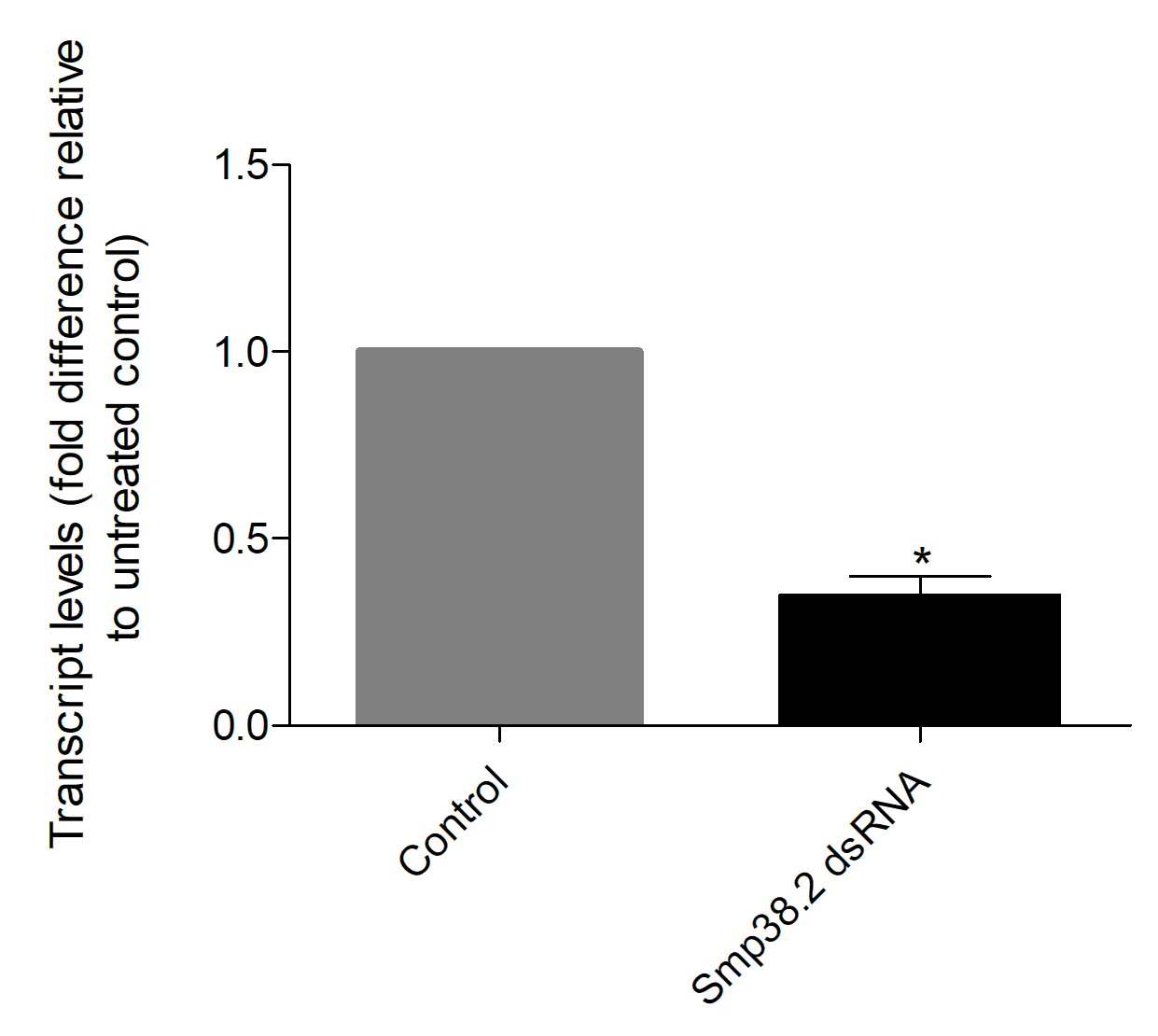

Supplement: Supplementary file 5 [file Image_5.TIF]

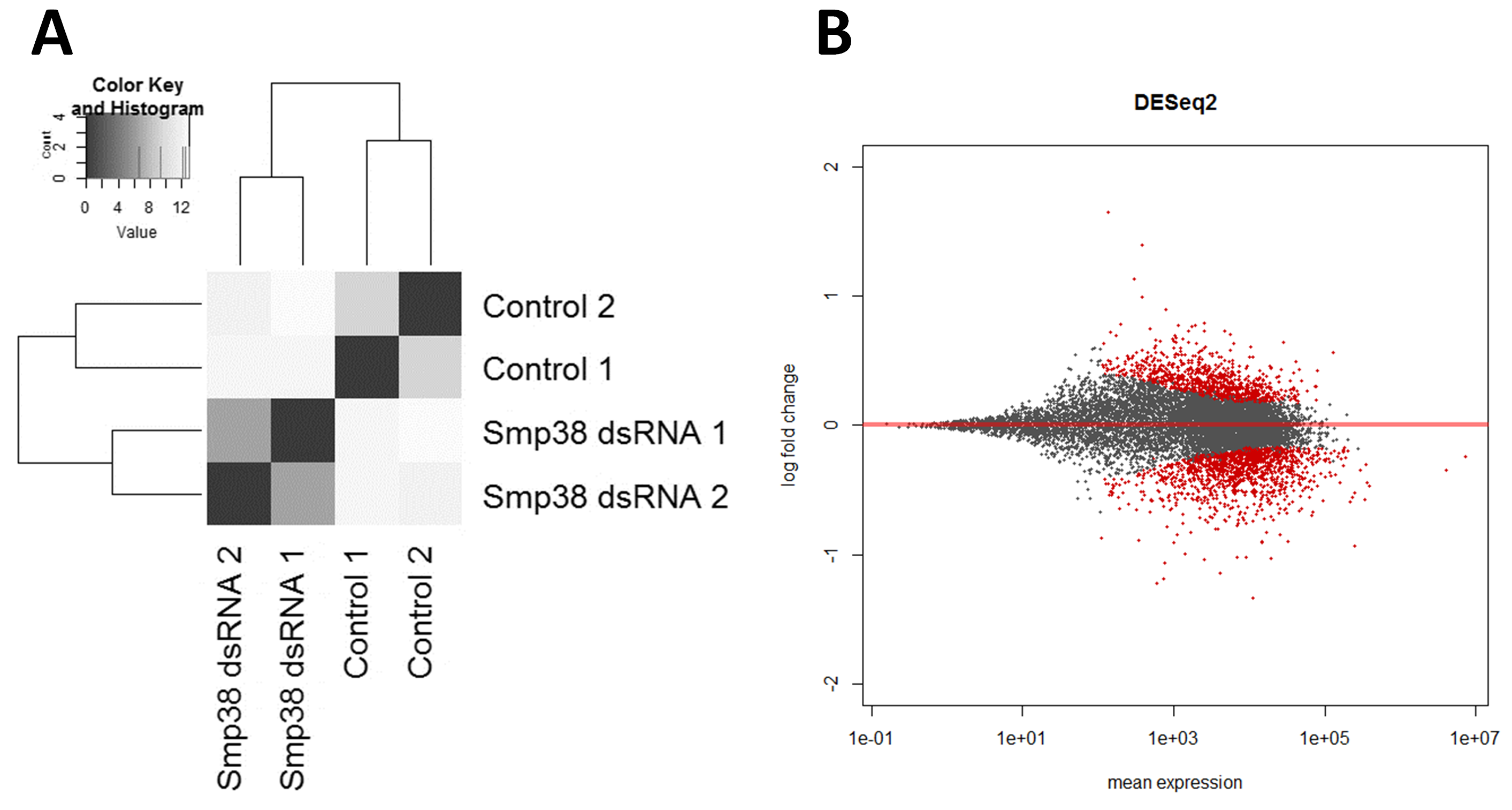

Supplement: Supplementary file 6 [file Image_6.TIF]

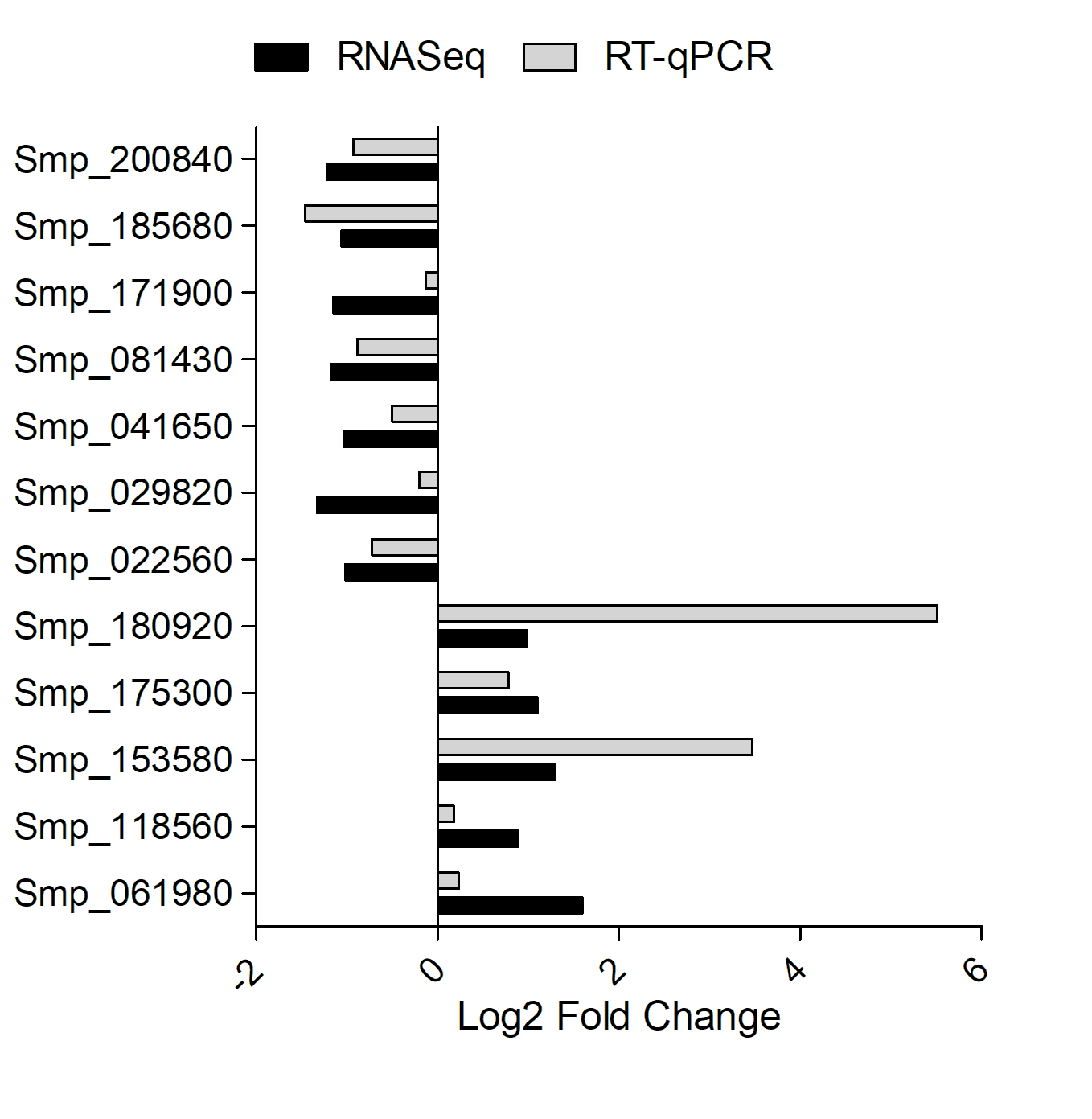

Supplement: Supplementary file 7 [file Image_7.TIF]

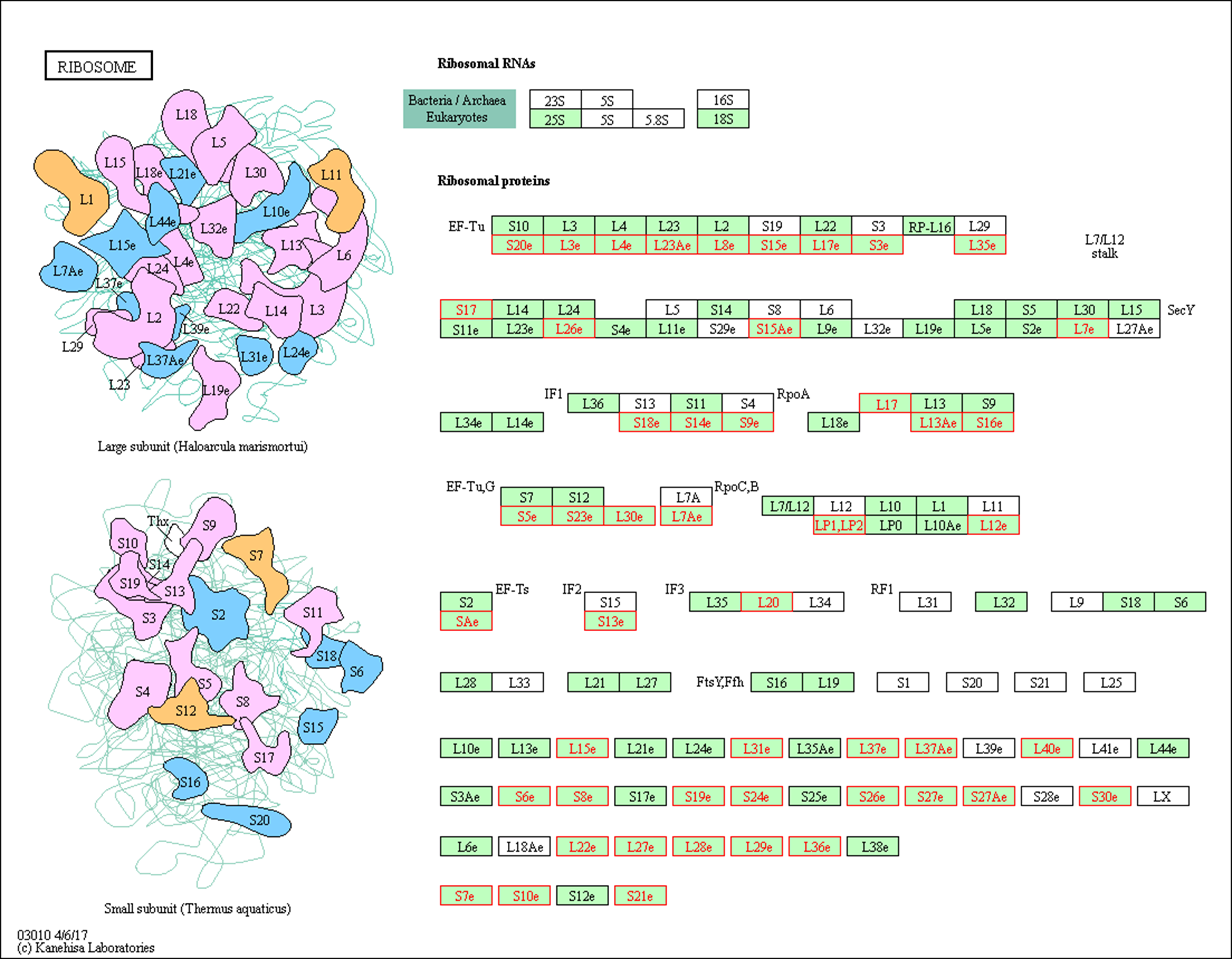

Supplement: Supplementary file 8 [file Image_8.TIF]

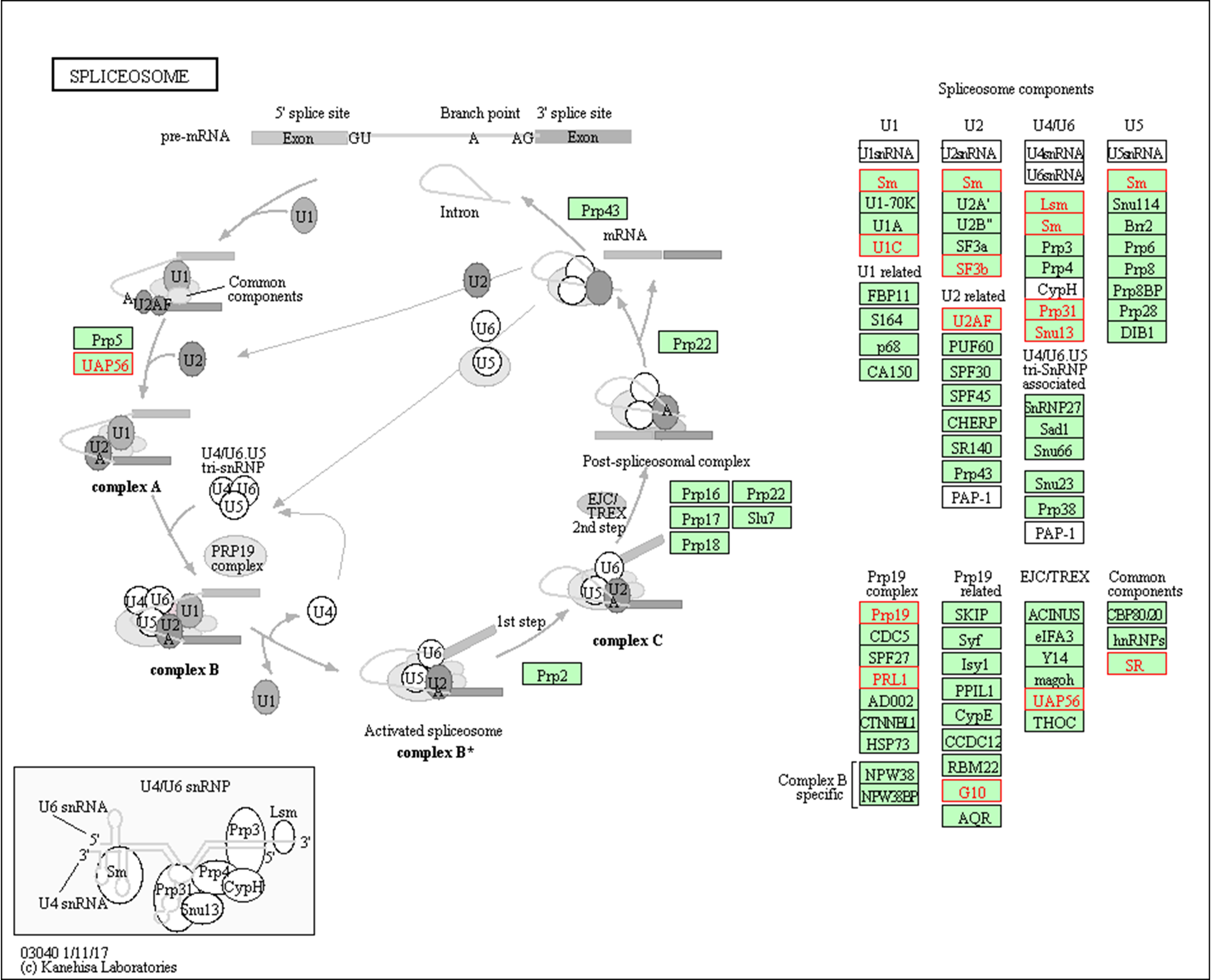

Supplement: Supplementary file 9 [file Image_9.TIF]

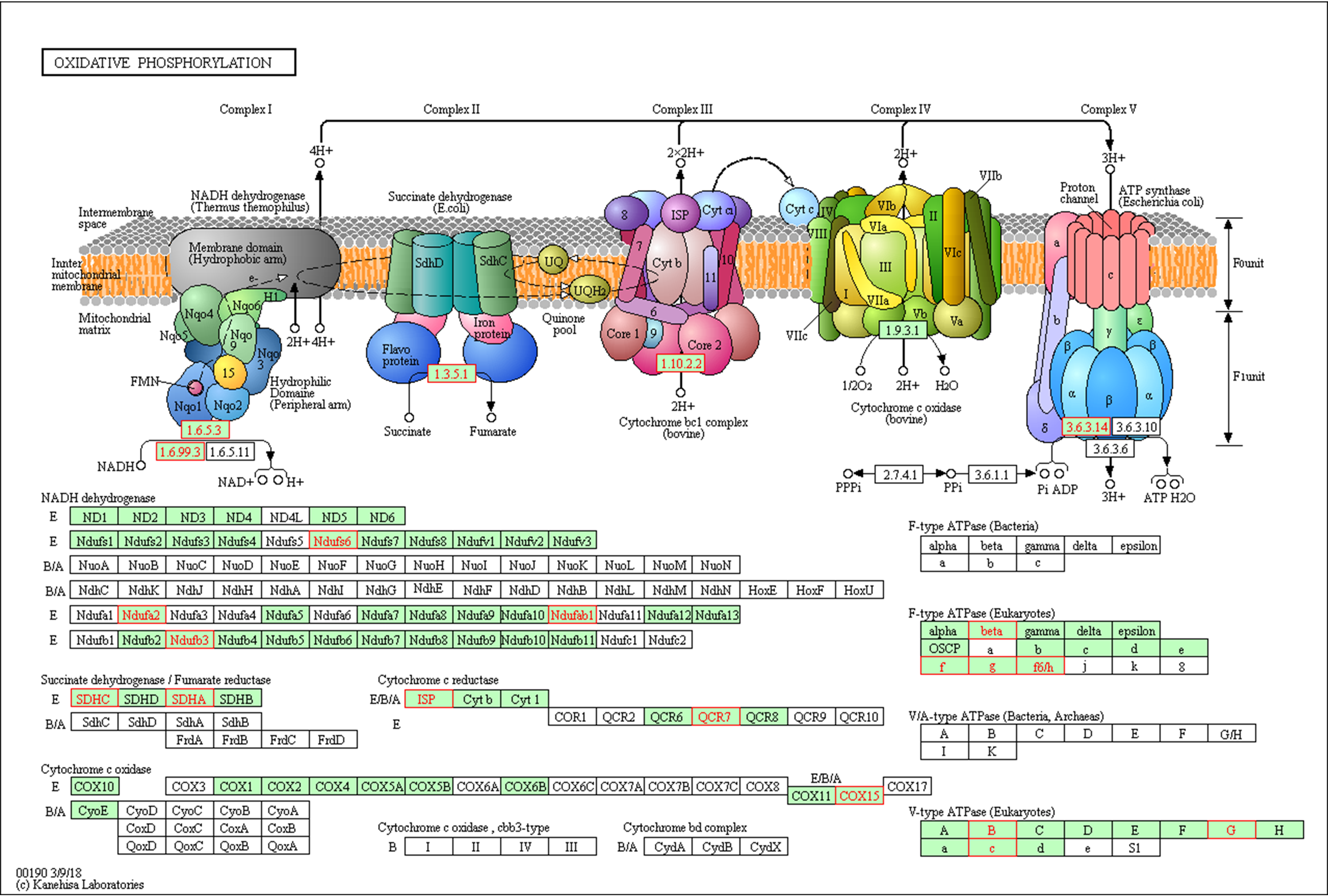

Supplement: Supplementary file 10 [file Image_10.TIF]

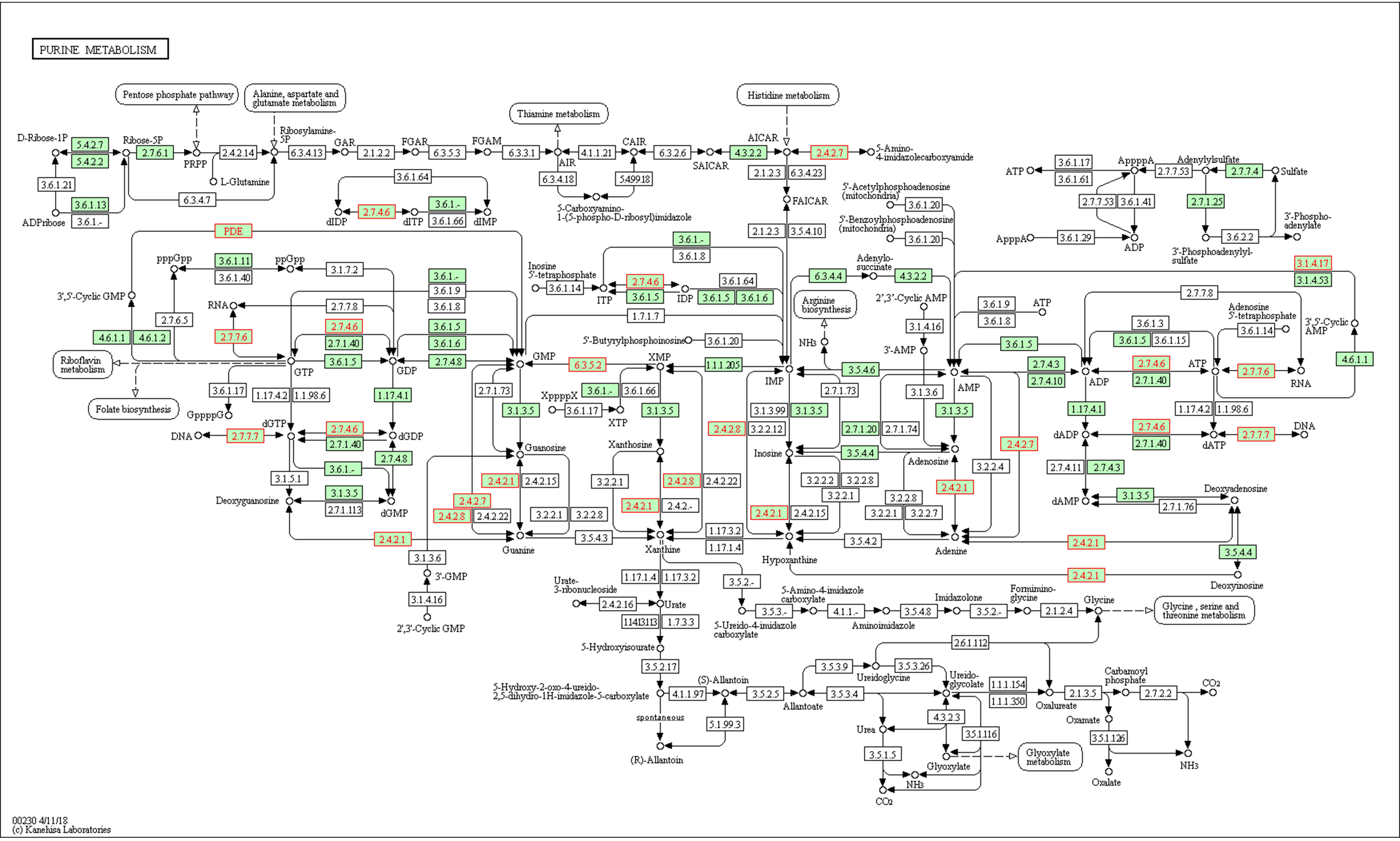

Supplement: Supplementary file 11 [file Image_11.TIF]
