# Supplementary material for: Smp38 MAP Kinase Regulation in Schistosoma mansoni: Roles in Survival, Oviposition, and Protection Against Oxidative Stress
Source: Front Immunol. 2019 Jan 24;10:21. doi: 10.3389/fimmu.2019.00021 (PMC6353789; doi:10.3389/fimmu.2019.00021)
Supplement: Supplementary file 13 [file Data_Sheet_1.PDF]

Supplementary Material

SMP38 MAP KINASE REGULATION IN *SCHISTOSOMA MANSONI*: ROLES IN SURVIVAL, OVIPOSITION AND PROTECTION AGAINST OXIDATIVE STRESS

Lívia das Graças Amaral Avelar[1,2¶], Sandra Grossi Gava[1,2¶], Renata Heisler Neves[3], Mercedes Carolina Soares da Silva[2], Neusa Araújo[2], Naiara Clemente Tavares[2], Assmaa El Khal[2], Ana Carolina Alves de Mattos[2], José Roberto Machado-Silva[3], Guilherme Oliveira[4]*, Marina de Moraes Mourão[2]*

*** CORRESPONDENCE:**
Marina de Moraes Mourão E-mail: marinamm@minas.fiocruz.br
Guilherme Oliveira E-mail: guilherme.oliveira@itv.org

Table S1. Primer sequences

**Complete coding sequence primers**			
**Target**	**Gene ID**	**Forward**	**Reverse**
Smp38 MAPK	Smp_133020	ATGATCGGGTATGGGGC	TCAAGCTGCATTTGTCTCTTC
		-	ACTGAATTGATGAATATGATTACCAC
**dsRNA primers**			
**Target**	**Gene ID**	**Forward**	**Reverse**
Smp38 MAPK	Smp_133020	taatacgactcactatagggACAGTATTGGCTTAATTCATCG	taatacgactcactatagggTGGATCATCGAATCTCTCTGC
		taatacgactcactatagggGTTGCAACTCGTTGGTATCG	taatacgactcactatagggTCGCGAATAAATCCACATGC
**Unspecific control**		**Forward**	**Reverse**
GFP	pCRII-GFP	taatacgactcactatagggTCTTCAAGTCCGCCATG	taatacgactcactatagggTGCTCAGGTAGTGGTTGTC
mCherry	GenBank AY678264	taatacgactcactatagggTGGTGAGCAAGGGCGA	taatacgactcactatagggTTACTTGTACAGCTCGTCC
**qPCR primers**			
**Target**	**Gene ID**	**Forward**	**Reverse**
Smp38 MAPK	Smp_133020	GCCCACGATAACAAATTAAACC	AAGGCATCAATCAAACAAACG
SmGCL	Smp_013860	TCCTGATAGTGGTGCTTCC	CCGTTCACGAATGTTACG
COX	Smp_900000	TACGGTTGGTGGTGTCACAG	ACGGCCATCACCATACTAGC

*The sequence of the T7 promoter is in lower case.

Table S2. Primer sequences used in RT-qPCR validation

Gene ID	Forward	Reverse
Smp_022560	CGATTATTCCAACAGCATGAGG	TCGGTTTATATGCGAGTGTATCG
Smp_029820	AGGCTTGACATCTGCTTGC	CCACAACTGATTGCTGAAGG
Smp_041650	AACGTTTGGTTCCCTCACC	TGACATAGAATTCGGTCACATCC
Smp_061980	GTCGTGAGCCTCTGTTCG	ACATCCCTATTGTCACTATTACTACC
Smp_081430	CGATGCAATAACAATCACACG	TGGTATGATGGCTACTAGTTATATGG
Smp_096750	CATTCCTGCCTAAGTGTTTACG	CGCATTCTGAAGTCCAAAGG
Smp_118560	CACCTGTTTTTAAAATTTGTCCCTA	AAATGCTCCATGAATATCGGTTA
Smp_153580	AGTGACTGATGAATCGACTAACTG	CTTATGGAAGACCAAGAAAGAAGC
Smp_171900	CTAAACCTGTTGTTGCACTTTCC	CTTGCGGATACGTTCCTATGG
Smp_175300	TGATTCATTTGGATCAATTAGGA	TTTGGAAATGTATTTGTTTGTATTG
Smp_180920	CCCTCTCCCTACCAAGTTT	GACTGACTGATTGAATAAGGGAATA
Smp_185680	CTTGAAATGTACCTTGCCATCC	AGAGAGAATGGGCTCAATGG
Smp_200840	CGCCTCCTTTACCATCACC	CCACTACCAACAACAACAAGC
Smp_202770	CGGAATACCTGCATCTTGACG	CAGTCAACGTGTTTGTTGTTGG

Table S3. Composition of raw reads in the control and Smp38 dsRNA libraries and align results against the reference genome of *S. mansoni*.

Library	Replicate	Total reads	Uniquely mapped reads number	Uniquely mapped reads %	Average mapped length	Number of reads mapped to multiple loci	% of reads mapped to multiple loci	Number of reads mapped to too many loci	% of reads mapped to too many loci	% of reads unmapped: too many mismatches	% of reads unmapped: too short	% of reads unmapped: other
Control	1	46650550	42272737	90.62	197.50	2944089	6.31	593304	1.27	0.00	1.39	0.41
	3	58555610	44470698	90.40	197.52	3049483	6.20	634067	1.29	0.00	1.73	0.38
Smp38.2 dsRNA	1	35140673	32022985	91.13	197.99	1949968	5.55	327681	0.93	0.00	2.10	0.29
	3	85264772	75741630	88.83	198.88	4921795	5.77	871989	1.02	0.00	4.02	0.36

Table S4. List of genes that are differentially regulated in the Smp38 dsRNA treatment.

EXCEL FILE

Supplementary Figure S1. Smp38 transcript levels in schistosomula before the infection of mice. Bar graph depicting the relative transcript levels of *Smp38* gene in schistosomula exposed to Smp38.1 (■) and Smp38.2 (■) dsRNAs prior to each mice infection. For each dsRNA tested, data are represented as mean fold-differences (+/- SE) relative to unspecific control (1.00 – dashed line). Transcript levels were determined by RT-qPCR and data analyzed using the $\Delta\Delta$Ct method (58), followed by statistical analysis using the Mann-Whitney test. * indicates that Smp38 transcript levels exhibit significantly difference relative to the controls; (* $p < 0.05$, ** $p < 0.005$, *** $p < 0.0005$), N = 3. Bars represent the standard error among replicates. S1 Figure.tif

Supplementary Figure S2. Assessment of the infectivity potential of miracidia from eggs recovered from mice infected with Smp38 knockdown parasites. Graph showing the percentage of snails shedding cercariae 30 days after exposure to miracidia originated from eggs recovered from mice previously infected with Smp38.1, Smp38.2 knockdown schistosomula or unspecific control. The percentage of infected snails is represented in grey (■) and not infected in black (■). S2 Figure.tif

Supplementary Figure S3. Smp38 transcript levels in miracidia recovered from eggs recovered from mice infected with Smp38 knockdown parasites. Bar graph depicting the relative Smp38 transcript levels in miracidia from eggs recovered from mice infected with schistosomula knockdown using Smp38.1 (■) or Smp38.2 (■) dsRNAs. Data are represented as mean fold-differences (+/- SE) relative to unspecific control (1.00 – dashed line). Transcript levels were determined by RT-qPCR and data analyzed using the $\Delta\Delta$Ct method (58), followed by statistical analysis using the Mann-Whitney test. Bars represent the standard error among replicates. S3 Figure.tif

Supplementary Figure S4. Viability curve of schistosomula after the exposure to SB 203580 inhibitor. Graph depicting the percentage of mortality of schistosomula exposed to different concentrations of SB 203580 inhibitor (■). Schistosomula were exposed to 10 µM, 25 µM, 50 µM and 100 µM SB 203580 and viability was assessed after 24 hours. Control (■) corresponds to parasites exposed to 0.02% v/v DMSO according to the inhibitor concentration tested. Significance was analyzed by Two-way ANOVA and the significant results were treated by the Bonferroni's test (*** p <0.001, N = 3). Bars represent the standard error among replicates. S4 Figure.tif

Supplementary Figure S5. Smp38 transcript levels in schistosomula exposed to Smp38 dsRNA before RNASeq experiments. Bar graph depicting relative transcript levels of Smp38 in schistosomula 2 days after exposure to Smp38.2 dsRNA. Data are represented as mean fold-differences (+/- SE) relative to untreated control. Transcript levels were determined by RT-qPCR and data analyzed using the $\Delta\Delta$Ct method (* p <0.05, N = 2). S5 Figure.tif

Supplementary Figure S6. Correlation plots of knockdown schistosomula libraries and the differentially expressed genes (DEGs). Correlation analysis of the RNASeq libraries constructed from Smp38 knockdown *S. mansoni* versus parasites from untreated control (A). MA plots of differentially expressed genes identified in Smp38 dsRNA libraries by employing DESeq2. Significantly altered genes were defined using a *p-adj* < 0.01 and are labeled in red. S6 Figure.tif

Supplementary Figure S7. RT-qPCR validation of differentially expressed genes in response to Smp38 knockdown. Validation of RNASeq approach using RT-qPCR. Twelve DEGs were selected from a range of upregulated and downregulated genes. Expression levels were quantified by RT-qPCR (■) and the results were compared to those obtained by the RNASeq (■) approach. S7 Figure.tif

Supplementary Figure S8. DEGs mapping to the ribosome structure KEGG pathway in Smp38 MAPK knockdown schistosomula. Genes down-regulated in the Smp38 MAPK knockdown schistosomula that were mapped to the KEGG pathway of ribosome (smm03010) are highlighted in red. S8 Figure.tif

Supplementary Figure S9. DEGs mapping to the spliceosome KEGG pathway in Smp38 MAPK knockdown schistosomula. Genes down-regulated in the Smp38 MAPK knockdown schistosomula that were mapped to the KEGG pathway of spliceosome (smm03040) are highlighted in red. S9 Figure.tif

Supplementary Figure S10. DEGs mapping to oxidative phosphorylation KEGG pathway in Smp38 MAPK knockdown schistosomula. Genes down-regulated in the Smp38 MAPK knockdown that were mapped to the KEGG pathway of oxidative phosphorylation (smm00190) are highlighted in red. S10 Figure.tif

Supplementary Figure S11. DEGs mapping to purine metabolism KEGG pathway in Smp38 MAPK knockdown schistosomula. Genes down-regulated by the Smp38 MAPK knockdown that were mapped to the KEGG pathway of purine metabolism (smm00230) are highlighted in red. S11 Figure.tif
